# Latent Cytomegalovirus (CMV) Infection Does Not Detrimentally Alter T Cell Responses in the Healthy Old, But Increased Latent CMV Carriage Is Related to Expanded CMV-Specific T Cells

**DOI:** 10.3389/fimmu.2017.00733

**Published:** 2017-06-26

**Authors:** Sarah E. Jackson, George X. Sedikides, Georgina Okecha, Emma L. Poole, John H. Sinclair, Mark R. Wills

**Affiliations:** ^1^Division of Infectious Diseases, Department of Medicine, University of Cambridge, Cambridge, United Kingdom

**Keywords:** human cytomegalovirus, immunology of aging, viral latency, human cytomegalovirus-specific T-cells, IFNγ production, cIL-10^+^CD4^+^ T cells, latent viral load

## Abstract

Human cytomegalovirus (HCMV) primary infection and periodic reactivation of latent virus is generally well controlled by T-cell responses in healthy people. In older donors, overt HCMV disease is not generally seen despite the association of HCMV infection with increased risk of mortality. However, increases in HCMV DNA in urine of older people suggest that, although the immune response retains functionality, immunomodulation of the immune response due to lifelong viral carriage may alter its efficacy. Viral transcription is limited during latency to a handful of viral genes and there is both an IFNγ and cellular IL-10 CD4^+^ T-cell response to HCMV latency-associated proteins. Production of cIL-10 by HCMV-specific CD4^+^ T-cells is a candidate for aging-related immunomodulation. To address whether long-term carriage of HCMV changes the balance of cIL-10 and IFNγ-secreting T-cell populations, we recruited a large donor cohort aged 23–78 years and correlated T-cell responses to 11 HCMV proteins with age, HCMV IgG levels, latent HCMV load in CD14^+^ monocytes, and T-cell numbers in the blood. IFNγ responses by CD4^+^ and CD8^+^ T-cells to all HCMV proteins were detected, with no age-related increase in this cohort. IL-10-secreting CD4^+^ T cell responses were predominant to latency-associated proteins but did not increase with age. Quantification of HCMV genomes in CD14^+^ monocytes, a known site of latent HCMV carriage, did not reveal any increase in viral genome copies in older donors. Importantly, there was a significant positive correlation between the latent viral genome copy number and the breadth and magnitude of the IFNγ T-cell response to HCMV proteins. This study suggests in healthy aged donors that HCMV-specific changes in the T cell compartment were not affected by age and were effective, as viremia was a very rare event. Evidence from studies of unwell aged has shown HCMV to be an important comorbidity factor, surveillance of latent HCMV load and low-level viremia in blood and body fluids, alongside typical immunological measures and assessment of the antiviral capacity of the HCMV-specific immune cell function would be informative in determining if antiviral treatment of HCMV replication in the old maybe beneficial.

## Introduction

A consequence of aging in the human population is a decline in immune function, often described as immune senescence, which includes a loss of adaptive immune cells and an increase in inflammatory cytokines resulting in dysregulation of the immune response (1). There is now evidence from a number of studies that, after the age of 65 years, the age-associated loss of immune function results in individuals becoming more susceptible to infectious diseases as well as increased morbidity and mortality from autoimmune disorders (2, 3). Infection with human cytomegalovirus (HCMV) is characterized by its lifelong persistence in the infected individual due, in part, to its ability to establish a latent infection in bone marrow stem cells and myeloid cells (4). Despite a robust immune response to the primary infection, the large number of immune evasion molecules encoded by HCMV allows it to establish its latent life cycle (5). Primary HCMV infection and reactivation from latency is generally well controlled in healthy individuals; however, when the immune system is compromised, or under developed, it can become a significant problem (6, 7). A potential impact of lifelong persistence of HCMV is its effect on the host immune response with aging. A number of longitudinal and population cohort studies have suggested that HCMV seropositivity was linked to age-related (i) increase in susceptibility to infections, (ii) poor response to vaccinations, and (iii) increased risk of all-cause mortality compared to age-matched HCMV seronegative individuals—which has been termed the immune risk phenotype (IRP) (8–13). Analysis of a number of large population cohorts recruited for cancer, dementia, and nutritional studies in the UK and USA have also shown a significant association between HCMV seropositivity and mortality from cardiovascular related disease (14–18). However, other studies have shown no such age-related correlation between HCMV seropositivity and declines in immune responses to either novel infections (19, 20) or responses to vaccination (21). Similarly, a study measuring frailty in older people saw a positive association with inflammatory cytokines but not HCMV infection (22) perhaps consistent with studies that have shown that rises in inflammatory cytokines in the serum of older donors is not primarily driven by HCMV (23).

It has been observed that infection with HCMV changes the composition of the CD4^+^ and CD8^+^ memory T cell repertoires; this includes an expansion of the T cell population, which have lost expression of the co-stimulatory molecules CD27 and CD28 but also show re-expression of CD45RA and co-expression of the carbohydrate HNK-1 (CD57) [reviewed in Ref. (24)]. Such T cells are considered to be a highly differentiated phenotype (25), and potentially dysfunctional as they often lose the ability to secrete cytokines and have limited proliferative capacity (11, 26). It has been suggested that expanded populations of highly differentiated T cells in HCMV seropositive older donors may be detrimental to the infected individual (27–29). However, such increases in these highly differentiated T cells is also observed in young HCMV positive individuals (30) and it is, also, now clear that these highly differentiated T cells are still functional and, with the correct co-stimulation, can proliferate (31, 32). Similarly, HCMV-specific T cells have been shown to produce multiple antiviral cytokines and have efficient cytotoxic capacity despite a highly differentiated phenotype (33–35). Furthermore, older HCMV seropositive individuals do not appear to suffer from overt HCMV disease from reactivating virus or HCMV re-infection which suggests that the immune response of older people retains the ability to control virus replication (36). Despite older HCMV seropositive donors having functional HCMV-specific immune responses, there does appear to be age-related increases in levels of viral DNA detectable in urine (36) and blood (37). This suggests that the immune response in older people may be altered, possibly due to lifelong carriage of the virus, and that immunomodulation of the HCMV-specific immune response, as either a direct consequence of the viral infection or bystander effects, results in reduced clearance of reactivating virus in older people (5).

Latent carriage of HCMV in CD34^+^ progenitor cells and their myeloid derivatives is characterized by repression of viral immediate Early (IE) gene transcription with a restricted gene expression profile, which cannot support production of infectious virus. A number of viral genes have been identified as being transcribed during HCMV latent infection, including UL138 (38), LUNA (latent undefined nuclear antigen; UL81-82as) (39, 40), US28 (41), UL111A (vIL-10) (42), and UL144 (43). Analysis of the secreted cellular proteins (cell secretome) of experimentally latently infected CD34^+^ and CD14^+^ cells have identified the induced expression of chemokines, which can recruit T cells as well as the cellular cytokines IL-10 and TGF-β, both of which can modulate the activity of T cells which have migrated to the environment surrounding the latent infection (44). HCMV-specific CD4^+^ T cells have been identified that either secrete cIL-10 or have a regulatory cell phenotype (45–49) and, in the mouse, it has been shown that CD4^+^ T regulatory cells (T_regs_) and IL-10 secretion can reduce viral clearance and increase persistence in murine cytomegalovirus (MCMV) (49, 50). Additionally, there is evidence that the frequency of HCMV-specific inducible T_regs_ is increased in older individuals (47), alongside an overall increase in frequency of T regulatory cells in old age (51, 52). Previously, we have identified CD4^+^ T cells specific for peptides to two of the latency-associated proteins, UL138 and LUNA, which secrete cIL-10 and also possess Th1 antiviral effector functions (53). We have also shown that the UL138-specific CD4^+^ T cells recognize experimentally latently infected CD14^+^ monocytes, secrete cIL-10, and suppress T cell function.

With these observations in mind, we hypothesized that the long-term carriage of HCMV could create an immunomodulatory environment to help prevent clearance of the virus by skewing the CD4^+^ T cell compartment toward a suppressive or regulatory cIL-10-producing phenotype. We also wanted to assess whether the same environment had an impact on the frequency of HCMV-specific CD8^+^ T cells within a large old aged donor cohort, who have carried HCMV for longer compared to younger seropositive donors. Additionally, within the study, we wanted to measure the levels of latent viral genome carriage and determine if infectious virus was detectable and relate this to changes in the T cell response. To address these questions, we conducted a study on a large healthy donor cohort, which encompassed a broad age range (23–78 years) of both HCMV seropositive and negative donors. We performed absolute cell counts, measured HCMV-specific antibody levels, assayed viral genome copy number in total peripheral blood and in CD14^+^ cells, as well as measuring the CD8^+^ specific production of IFNγ and CD4^+^ specific production of IFNγ and IL-10 in response to stimulation by overlapping peptide pools to 11 HCMV proteins (5 latency associated and 6 lytic only expressed proteins). The study group exhibited typical age-related decline in both absolute CD4^+^ and CD8^+^ naïve T cell numbers and HCMV seropositive donors had increased absolute numbers of T cells with a differentiated phenotype compared to seronegative donors. We did not see an inversion of the CD4:CD8 ratio within this donor cohort, a characteristic associated with the IRP, although CD4:CD8 ratio was decreased in HCMV seropositive donors compared to seronegative. In contrast to studies in other donor cohorts, we did not see an age-related expansion of the HCMV IgG response or an influence of donor age on either the breadth or magnitude of the T cell responses (24, 54). We detected both CD4^+^ and CD8^+^ specific IFNγ responses to all 11 HCMV proteins analyzed and also detected more limited CD4^+^ specific IL-10 responses to the same proteins, and we also confirmed our previous observations that CD4^+^ specific IL-10 responses are more common toward latency-associated proteins. We were able to detect latent HCMV genomes in isolated peripheral blood CD14^+^ monocytes in 45% of donors but, in contrast to previous reports (54), we did not observe an increase in HCMV copy number in donors aged over 70 years old. Importantly, we did see a significant association between the levels of HCMV detected in CD14^+^ monocytes and both the breadth and magnitude of the CD8^+^ T cell responses to HCMV proteins, irrespective of donor age. Overall it is our opinion that larger latent HCMV reservoirs will lead to increased HCMV reactivation and dissemination events, which in normal healthy individuals will stimulate secondary HCMV-specific T cell responses, thus driving increases in T cell frequency and differentiation status.

## Materials and Methods

### Ethics and Donor Cohort Information

The study donor cohort was recruited by the National Institute of Health Research Cambridge BioResource, using their Biobank of volunteers, who predominantly are local to Cambridge or live in the East Anglian Region of the UK. Ethical approval was obtained from University of Cambridge Human Biology Research Ethics Committee. Informed written consent was obtained from all donors in accordance with the Declaration of Helsinki (HBREC.2014.07). Known HCMV seropositive and seronegative donors were recruited in three age groups; young (18–40 years), middle (41–64 years), and old (65^+^ years) were included in this study. Volunteers being treated with oral or intravenous immunomodulatory drugs (including steroids, tacrolimus, cyclosporins, azathioprines, mycophenolate, methotrexate, rituximab, and cyclophosphamide) within the last 3 months, undergoing injected rheumatoid arthritis treatment including anti-TNFα agents and anyone actively, or within the last 24 months, being treated with cancer chemotherapy were excluded from the study. 119 HCMV seropositive and seronegative donors were included in this study, the age range of the recruited donor cohort was 23–76 years, 70 donors were female and 49 donors were male. Further characteristics of the studied donor cohort are detailed in Table 1. In total, a 50 ml peripheral blood sample was collected from each donor, comprising 1.2 ml clotted blood, 1.2 ml EDTA treated blood, and 47.6 ml lithium heparin treated blood samples.

**Table 1:** ARIA cohort donor characteristics.

		All ages		Young (<40 years)	Middle (41–64 years)	Old (>65 years)
			
		Human cytomegalovirus (HCMV) +ve	HCMV −ve	HCMV +ve	HCMV +ve	HCMV +ve
Donors (*n*)	All	105	14	33	31	41
	M	44	5	14	14	16
	F	61	9	19	17	25

Age (years) (mean ± SD)	All	54.4 ± 15.6	51.4 ± 14.4	34.6 ± 5.1	54.5 ± 6.0	70.2 ± 3.1
	M	54.3 ± 16.1	46.2 ± 12.7	34.7 ± 5.5	53.9 ± 6.5	71.7 ± 2.8
	F	54.5 ± 15.3	54.3 ± 14.4	34.6 ± 4.8	54.9 ± 5.5	69.3 ± 2.8

HCMV IgG (ISR) (mean ± SD)	All	3.78 ± 1.28	0.28 ± 0.14	3.66 ± 1.32	3.81 ± 0.99	3.85 ± 1.42
	M	3.67 ± 0.95	0.25 ± 0.10	3.15 ± 0.74	4.06 ± 0.74	3.78 ± 1.06
	F	3.86 ± 1.47	0.29 ± 0.15	4.03 ± 1.51	3.60 ± 1.12	3.90 ± 1.61

HCMV DNAemia (copies/ml blood) (mean ± SD)	All	2.6 ± 26.7^a^	Undetected	Undetected	Undetected	6.7 ± 42.4^a^
	M	6.3 ± 41.0^a^	Undetected	Undetected	Undetected	17.2 ± 66.6^a^
	F	Undetected	Undetected	Undetected	Undetected	Undetected

CD4:8 ratio (mean ± SD)	All	2.25 ± 1.61	3.60 ± 1.80	2.04 ± 0.85	1.96 ± 0.85	2.63 ± 2.29
	M	2.10 ± 1.00	4.00 ± 2.10	1.80 ± 0.70	2.00 ± 1.00	2.30 ± 1.20
	F	2.40 ± 1.90	3.40 ± 1.50	2.20 ± 0.90	1.90 ± 0.70	2.90 ± 2.80

*Summary of the number of donors and age ranges, serum HCMV IgG levels [immune status ratio (ISR)], blood HCMV DNA copies and the CD4:CD8 ratio (generated from absolute count data)*.

*^a^HCMV DNAemia detected in n = 1 old male donor*.

### Peripheral Blood Mononuclear Cell (PBMC) Isolation

Peripheral blood mononuclear cells were isolated from the heparinized blood samples using Lymphoprep (Axis-shield, Oslo, Norway) density gradient centrifugation.

### Absolute Count Protocol

50 µl of the EDTA treated whole blood sample was transferred to Becton Dickinson Trucount tubes (BD Biosciences, Oxford, UK) and stained with a pre-mixed antibody cocktail containing CD45-VioBlue, CD3-VioGreen (Miltenyi Biotec, Bisley, UK.), CD4-Brilliant Violet 605, CD8-PerCP-Cy5.5, CD28-PE, CD27-APC-Cy7, CD45RA-FITC, CD25-APC, and CD127-PE-Cy7 (BioLegend, San Diego, CA, USA). Following staining, the red blood cells were lysed and the cells fixed using FACS Lysing solution (BD Biosciences). The samples were stored at −80°C until acquisition (55). Samples were acquired on a LSR Fortessa (BD Biosciences) along with Fluorescence Minus One (FMO) controls using FACS Diva software (BD Biosciences). Samples were then analyzed using FlowJo software (Treestar, OR, USA), first the trucount bead population was identified and then the trucount bead negative population (i.e., cells) were analyzed by gating for single cells, then CD45^hi^ lymphocytes, CD3^+^ T cells, CD4^+^ and CD8^+^ expressing cells. The CD4^+^ and CD8^+^ T cell populations were further subdivided into four memory populations defined by expression of CD27 and CD45RA, and four differentiation populations defined by expression of CD27 and CD28 were identified, and in CD4^+^ T cells, a T_reg_ population defined as CD25^hi^ and CD127^lo^ were identified, gate and quadrant positions were identified using the FMO controls. A representative gating strategy and the formula used to calculate the absolute cell counts is illustrated in Figure S1 in Supplementary Material, the event number for all populations, and trucount beads were exported to an excel sheet where the number of cells per microliter of blood for each T cell subset was calculated according to manufacturer instructions.

### HCMV IgG Antibody Levels Protocol

Human cytomegalovirus serostatus was confirmed using serum from the clotted blood sample and HCMV IgG levels determined using an IgG enzyme-linked immunosorbent (EIA) assay, HCMV Captia (Trinity Biotech, Didcot, UK) following manufacturer’s instructions, on serum derived from clotted blood samples. The EIA assay is semi-quantitative, containing negative, positive and calibrator controls which allow the computation of an immune status ratio (ISR) value for the amount of HCMV IgG present in the sample. In addition to the manufacturer controls and quality control protocols, a known positive serum sample was also run to check inter-assay variability was acceptable.

### HCMV ORF Peptide Mixes

8 HCMV ORF encoded proteins [UL55 (gB), UL82 (pp71), UL122 (IE2), UL123 (IE1), US3, UL138, US28, and UL111A(vIL-10)] were selected and peptide libraries comprising consecutive 15mer peptides overlapping by 10 amino acid were synthesized by ProImmune PEPScreen (Oxford, UK) from sequences detailed in the Sylwester et al. study (56). A further 3 HCMV ORF encoded proteins [UL83 (pp65), UL144 (which incorporated known strain variants) and LUNA (UL81-82as)] 15mer peptide libraries were synthesized by JPT Peptide Technologies GmbH (Berlin, Germany). The individual lyophilized peptides from each ORF library were reconstituted and used as previously described (57).

### Depletion of CD4^+^ and CD8^+^ T Cells from PBMCs

Peripheral blood mononuclear cells were depleted of either CD4^+^ or CD8^+^ T cells by MACS using anti-CD4^+^ or anti-CD8^+^ direct beads (Miltenyi Biotec), according to manufacturer’s instructions, and separated on either LS columns (Miltenyi Biotec) or by using an AutoMACS Pro (Miltenyi Biotec). Efficiency of depletion was determined by staining cells with a CD3-FITC, CD4-PE, and CD8-PerCPCy5.5 antibody mix (all BioLegend) and analyzed by flow cytometry. Depletions performed in this manner resulted in mean 3.8% residual CD8^+^ T cells and 8.6% residual CD4^+^ T cells (from *n* = 61 donors).

### Dual FluoroSpot Assays

2 × 10^5^ PBMC depleted of either CD8^+^ or CD4^+^ T cells suspended in X-VIVO 15 (Lonza, Slough, UK) supplemented with 5% Human AB serum (Sigma Aldrich) were incubated in precoated FluoroSpot plates [Human IFNγ and IL-10 FluoroSpot (Mabtech AB, Nacka Strand, Sweden)] in triplicate with ORF mix peptides (final peptide concentration 2 μg/ml/peptide) and an unstimulated and positive control mix [containing anti-CD3 (Mabtech AB), Staphylococcus Enterotoxin B, Phytohemagglutinin, Pokeweed Mitogen, and Lipopolysaccharide (all Sigma-Aldrich)] at 37°C in a humidified CO_2_ atmosphere for 48 h. The cells and medium were decanted from the plate and the assay developed following the manufacturer’s instructions. Developed plates were read using an AID iSpot reader (Oxford Biosystems, Oxford, UK) and counted using AID EliSpot v7 software (Autoimmun Diagnostika GmbH, Strasberg, Germany) using distinct counting protocols for IFNγ and IL-10 secretion. Donor results were discounted from further analysis if there was greater than 1,000 spot forming units (sfu) background secretion of IFNγ or IL-10 in the unstimulated wells, additionally the sfu response in the positive control wells had to be at least 100 sfu (IFNγ) or 50 sfu (IL-10) greater than the background sfu. All data were then corrected for background cytokine production and the positive response cutoff for IFNγ and the IL-10 responses was determined by comparing the distribution of the responses from HCMV seropositive and seronegative donors to all HCMV proteins and the positive control. This analysis determined that the positive response for IFNγ and IL-10 was greater than 100 sfu/million, this threshold is indicated in Figures 3A, 4A and 5A (dashed line).

### Measurement of HCMV DNAemia in Whole Blood

A 1 ml EDTA treated whole blood sample was stored at −20°C for each donor. DNA was isolated from the whole blood sample using the QIAamp DNA Blood Midi Kit (Qiagen, Manchester, UK) following the manufacturer’s instructions. Extracted DNA samples were stored at −20°C until required. The detection of HCMV by real-time quantitative PCR method using the StepOne Real-Time PCR system (Applied Biosystems, ThermoFisher Scientific) was performed using a method adapted from Ref. (58). Real-time amplification of HCMV DNA used glycoprotein B-specific primers [5′-GAGGACAACGAAATCCTGTTGGGCA-3′ [gB1] and 5′-GTCGACGGTGGAGATACTGCTGAGG-3′ [gB2] (59)], and detection with a TaqMan probe [5′ 6-FAM- CAATCATGCGTTTGAAGAGGTAGTCCA-BHQ1 3′ [gBP3] (58)] mixed with ABI Universal Mastermix (Applied Biosystems, ThermoFisher Scientific), the final assay volume was 25 µl, which includes a 5 µl donor or control sample. PCR cycling conditions were 2 min at 50°C, 10 min at 95°C, and 45 cycles of 15 s at 95°C and 60 s at 60°C, all donor samples were screened in duplicate with a high (50,000 copies/ml) and low (500 copies/ml) positive control samples [whole EDTA treated blood spiked with HCMV genomes from the World Health Organization (WHO) international standard (60) (National Institute for Biological Standards and Control, Potters Bar, UK)], run in triplicate. Samples with detectable HCMV DNA were repeated in triplicate in a real-time amplification including a standard curve in triplicate of 1–10^4^ HCMV genomes (WHO International Standard) in addition to the high and low positive controls. The HCMV DNA load was calculated using the StepOne Software (Applied Biosystems, ThermoFisher Scientific) and reported as HCMV copies per milliliter blood.

### Latent Viral Load Digital PCR

CD14^+^ monocytes were extracted using CD14^+^ magnetic beads and MS columns (Miltenyi Biotec) from PBMC isolated from 20 ml of heparinized peripheral blood in a HCMV clean facility. The monocytes were enumerated, dry pelleted, and stored at −80°C prior to DNA extraction. DNA was extracted from the cell pellet in a 1:1 mixture of PCR solutions A (100 mM KCl, 10 mM Tris–HCl pH 8.3, and 2.5 mM MgCl_2_) and B (10 mM Tris–HCl pH 8.3, 2.5 mM MgCl_2_, 1% Tween 20, 1% Non-idet P-40, and 0.4 mg/ml Proteinase K) at a final concentration equivalent to 10,000 cells/μl, for 60 min at 60°C followed by a 10 min 95°C incubation (61), extracted DNA samples were stored at −80°C until required. Measurement of HCMV DNA in extracted CD14^+^ cells was assessed using a droplet digital PCR method (54). Using the QX200 droplet digital PCR system (Bio-Rad, Watford, UK), a reaction mixture containing 2 µl of donor CD14^+^ DNA (equivalent to 20,000 cells) or positive control sample was mixed with PCR grade water, 2× digital droplet PCR (ddPCR) supermix for probes (Bio-Rad), FAM labeled HCMV primer and probe (from Human CMV HHV5 kit for qPCR using a glycoprotein B target, PrimerDesign, Southampton, UK) and HEX labeled RPP30 copy number assay for ddPCR (Bio-Rad). Droplets were generated with droplet generation oil (Bio-Rad) in the QX200 droplet generator (Bio-Rad), then the sample was loaded into a 96-well PCR plate (Eppendorf, Stevenage, UK), sealed with a PX1 PCR Plate sealer (Bio-Rad) and PCR amplification was performed using a C1000 Touch Thermocycler (Bio-Rad), for 10 min at 95°C followed by 40 cycles of 30 s at 94°C and 60 s at 60°C. Following PCR amplification, the PCR plate was loaded onto the QX200 Droplet Reader (Bio-Rad) where the presence or absence of PCR product in each droplet was read and analyzed by QuantaSoft software (Bio-Rad), which gives the result of the number of virus copies per microliter of PCR reaction. All donor CD14^+^ DNA samples were run in either quadruplicate or triplicate. The RPP30 copy number primer probe enabled the determination of the cell number included in the reaction and the HCMV viral load number was adjusted according to this and expressed as HCMV copies per million CD14^+^ cells.

### Statistics

Statistical analysis was performed using GraphPad Prism version 6.00 for Windows (GraphPad Software, San Diego, CA, USA). Correlation was assessed by Pearson or Spearman correlation according to the distribution of the data. Multiple data sets groups were compared using a one-way ANOVA Kruskal–Wallis test with *post hoc* Dunn’s multiple comparisons or selected Mann–Whitney *U* comparisons using an adjusted *p* value (*p* ≤ 0.05/*n* comparisons) to correct for multiple testing false discovery.

## Results

### Characterization of the ARIA Study Donor Cohort

To determine whether long-term carriage of HCMV alters the HCMV-specific T cell response, with respect to cytokine secretion or state of T cell differentiation, and whether any identified changes impact on latent HCMV viral carriage and/or levels of HCMV IgG, we designed an age cross-sectional study. Donors were placed into three age groups: young (age ≤40 years), middle aged (age 41–64 years), and old (age ≥65 years) and also grouped on the basis of their HCMV serostatus. Potential donors were excluded from the study if they were currently taking, or had taken in the previous 3 months, any immunomodulatory or monoclonal antibody treatments or if they were currently cancer sufferers or had any form of cancer in the previous 24 months. In total, 119 individuals from the three age groups were included in this analysis: age range, virological and immunological parameters (HCMV IgG levels, HCMV DNA copies per milliliter whole blood and the CD4:CD8 ratio) for the donor cohort are detailed in Table 1. Correlation of the levels of HCMV IgG (ISR) (summarized for the three age groups in Table 1) within HCMV seropositive (HCMV +ve) donors with age did not show a significant accumulation with age {Pearson *r* = 0.1012 [95% confidence interval (CI): −0.0923, 0.2873], *p* = 0.3043}. Neither was there a significant decrease in the CD4:CD8 ratio within the HCMV +ve donor group with age [Spearman *r*_s_ = 0.08563 (95% CI: −0.1135, 0.2781), *p* = 0.3851].

**Figure 1:**
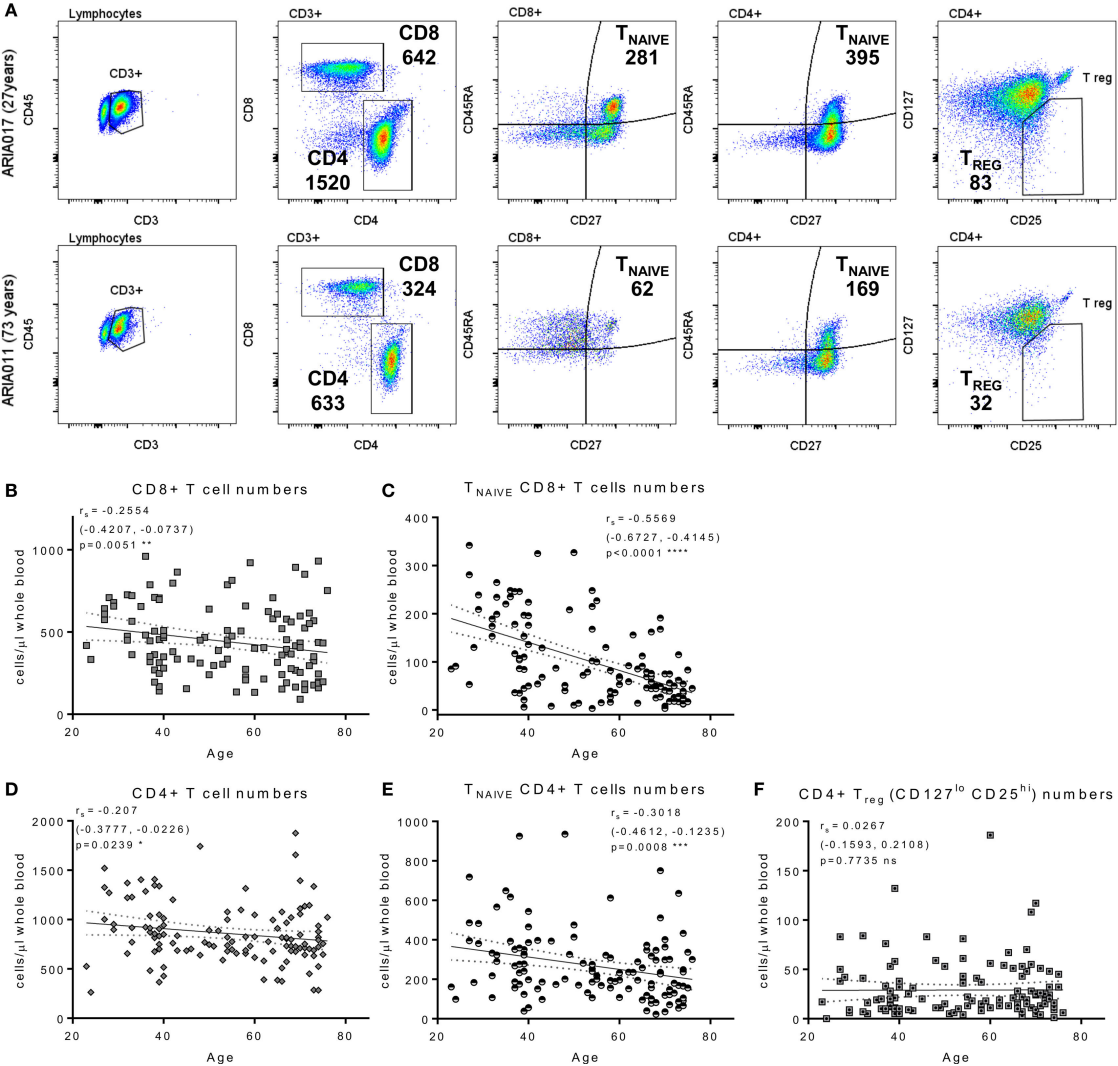
Impact of aging on T cell numbers. EDTA treated whole blood was stained with a panel of phenotyping antibodies in order to enumerate CD4^+^ and CD8^+^ T cells and their subsets. Representative dot plots from a young and old donor showing CD4^+^ and CD8^+^ T cell gates, naïve T cells subset were defined by CD27^+^ and CD45RA^+^ (T_NAIVE_) expression, and CD4^+^ T regulatory cells (T_reg_—CD25^hi^, CD127^lo^); the number of cells per microliter of whole blood present for each gated population of interest are also indicated **(A)**. Graphs illustrating the numbers of total CD8^+^ T cells **(B)**, T_NAIVE_ CD8^+^ T cells **(C)**, total CD4^+^ T cells **(D)**, T_NAIVE_ CD4^+^ T cells **(E)**, and CD4^+^ T_reg_ cells **(F)** of the entire ARIA cohort (*n* = 119) correlated to donor age. The relationship of T cell subset numbers with donor age was analyzed using Spearman rank correlation with the results indicated on each graph [r_s_ (95% confidence interval) and *p* value]. There was a significant decrease in total and T_NAIVE_ CD4^+^ and CD8^+^ T cells with age, CD4^+^ T_reg_ numbers showed no significant difference.

**Figure 2:**
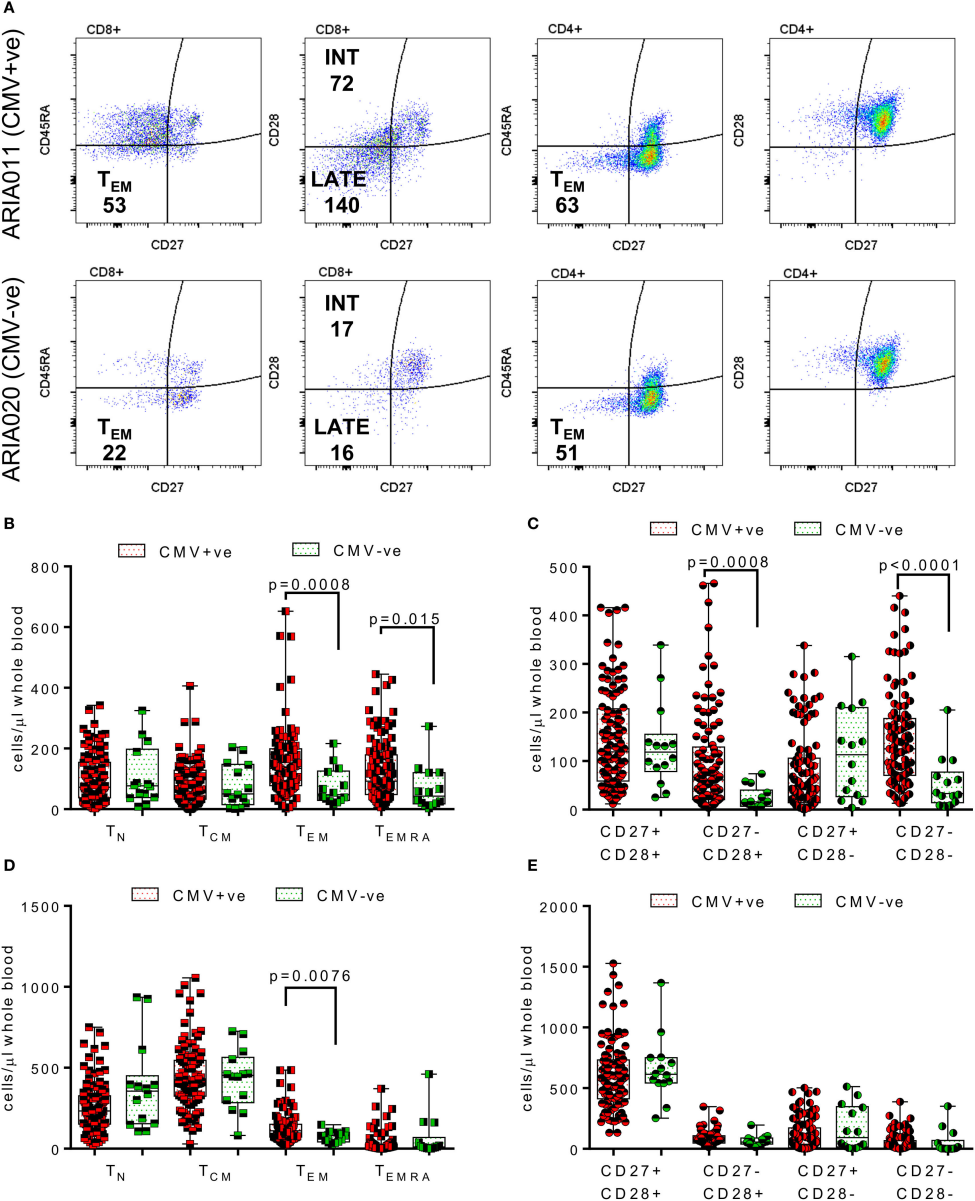

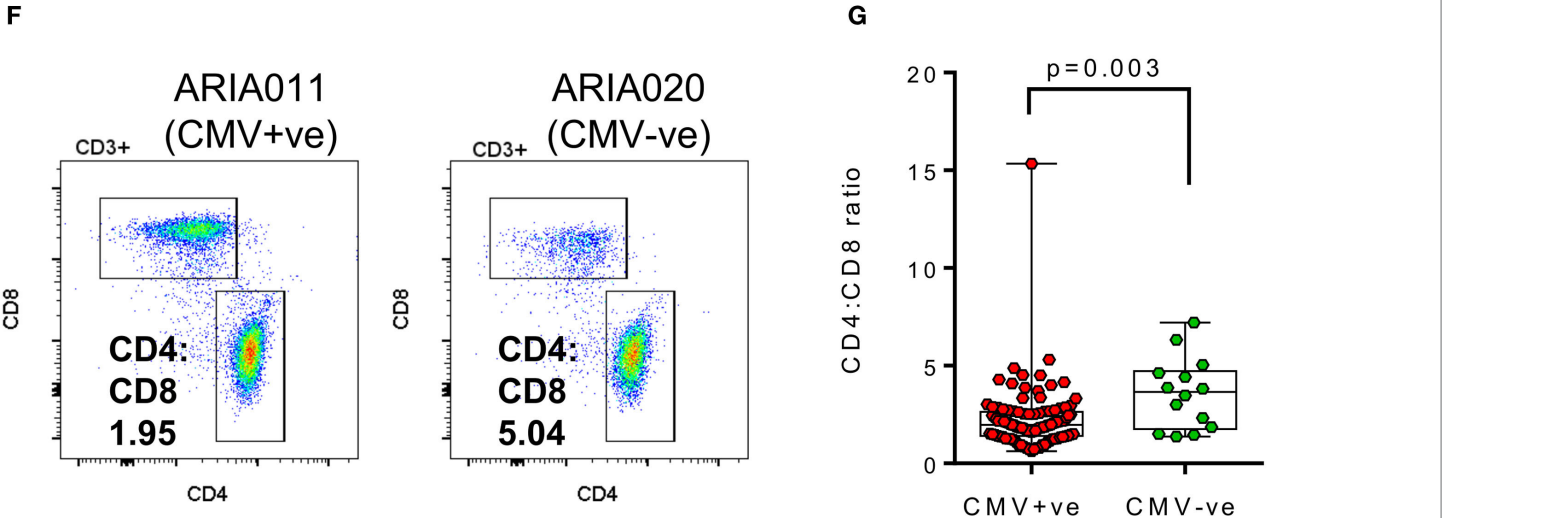
Impact of human cytomegalovirus (HCMV) carriage on T cell numbers. EDTA treated whole blood was stained with a panel of phenotyping antibodies in order to enumerate CD4^+^ and CD8^+^ T cells and their subsets. Representative dot plots from a HCMV seropositive (HCMV +ve) and HCMV seronegative (HCMV-ve) age-matched donors are illustrated showing the memory (as defined by CD27 and CD45RA expression) and differentiation level (as defined by CD27 and CD28) phenotype of both CD4^+^ and CD8^+^ T cells; the number of cells per microliter of whole blood for effector memory (T_EM_—CD27^−^CD45RA^−^) CD4^+^ and CD8^+^ T cells and Intermediate (INT—CD27^−^CD28^+^) and Late (LATE—CD27^−^CD28^−^) differentiated CD8^+^ T cells are shown **(A)**. Box and whisker plots comparing cell numbers of the memory **(B,D)** and differentiation phenotypes **(C,E)** of CD8^+^ T cells and CD4^+^ T cells, respectively, between HCMV +ve (red) and HCMV-ve (green) donors are shown. The differences between the two groups were analyzed by a Kruskal–Wallis one-way ANOVA test with *post hoc* Mann–Whitney *U*-test performed with significant results set as *p* ≤ 0.015 shown on each graph. A representative CD4 vs CD8 dot plot from the same donors with their respective CD4:CD8 ratio indicated are shown **(F)**, the comparison of CD4:CD8 ratios for all seropositive vs seronegative donors are also shown **(G)** with the significant decrease in the CD4:CD8 ratio in HCMV positive donors indicated (Mann–Whitney test).

The composition of the CD8^+^ and CD4^+^ T cell compartments, in whole blood isolated directly *ex vivo*, were enumerated and compared between donor age and HCMV serostatus. Figure 1 summarizes the impact of increasing age on T cell numbers in the entire donor cohort. This analysis shows that both CD8^+^ and CD4^+^ T cell numbers significantly decrease with age (Figure 1B, Spearman *r*_s_ = −0.255, *p* = 0.005 and Figure 1D, Spearman *r*_s_ = −0.207, *p* = 0.024, respectively), which was likely due to the significant loss of naïve CD8^+^ and CD4^+^ T cells (Figures 1C,E) with no corresponding increase in numbers of memory T cell populations (Figure S2 in Supplementary Material). Enumeration of CD4^+^ T regulatory cells present in the peripheral blood of all donors, based on the expression of CD127 and CD25 (62), showed that there was no effect of age on the size of this cell population (Figure 1F). When comparing the impact of HCMV infection, in donors of all ages, on the numbers of differentiated T cell subsets [representative donor phenotype staining is shown (Figure 2A)], we observed a significant expansion of the effector memory (T_EM_—CD27^−^CD45RA^−^) population in both CD8^+^ (Figure 2B) and CD4^+^ T cells (Figure 2D). Within CD8^+^ T cells only, we also saw a significant increase in the highly differentiated T_EMRA_ (CD27^−^CD45RA^+^) and CD27^−^CD28^−^ (LATE) populations (Figures 2B,C), which was not observed in CD4^+^ T cells (Figures 2D,E). A key component of the IRP, which is associated with HCMV infection, is the inversion of the CD4:CD8 ratio (<1), we only saw this phenomenon in 10% of the seropositive donor group. However, we observed that overall the CD4:CD8 ratio was significantly decreased in HCMV seropositive donors compared to seronegatives (Figures 2F,G).

### Magnitude and Breadth of T Cell Responses to HCMV Proteins Remain Stable with Donor Age

To establish whether HCMV latent and lytic protein specific T cells are maintained and are functional during long-term carriage of the virus, we analyzed T cell responses to five viral genes known to be expressed during HCMV latent infection: UL138 (38), LUNA (39, 40), US28 (41), UL111A (vIL-10) (42), and UL144 (43), two of which (UL138 and LUNA), we have previously shown elicit both an IFNγ and IL-10 CD4^+^ T cell response (53). We also wanted to measure the range of T cell responses in a large donor cohort to a number of viral proteins expressed during lytic infection; we have previously identified both CD4^+^ and CD8^+^ T cells producing IFNγ from many donors to six HCMV lytic proteins pp65, IE1, IE2, gB, pp71, and US3 (57, 63). Using FluoroSpot methodology, we were able to measure CD8^+^ T cell IFNγ responses and both IFNγ and IL-10 CD4^+^ T cell responses to overlapping peptide pools of these 11 HCMV proteins. Both HCMV seropositive and seronegative donors of all ages were included in these antigen-specific screens and, after discounting samples following quality control [high spontaneous cytokine spot forming unit (sfu) counts in unstimulated wells or failure of positive control stimulation], 98 donors were included in the CD8^+^ T cell analysis, 99 donors in the CD4^+^ T cell IFNγ analysis, and 73 donors in the CD4^+^ T cell IL-10 analysis.

**Figure 3:**
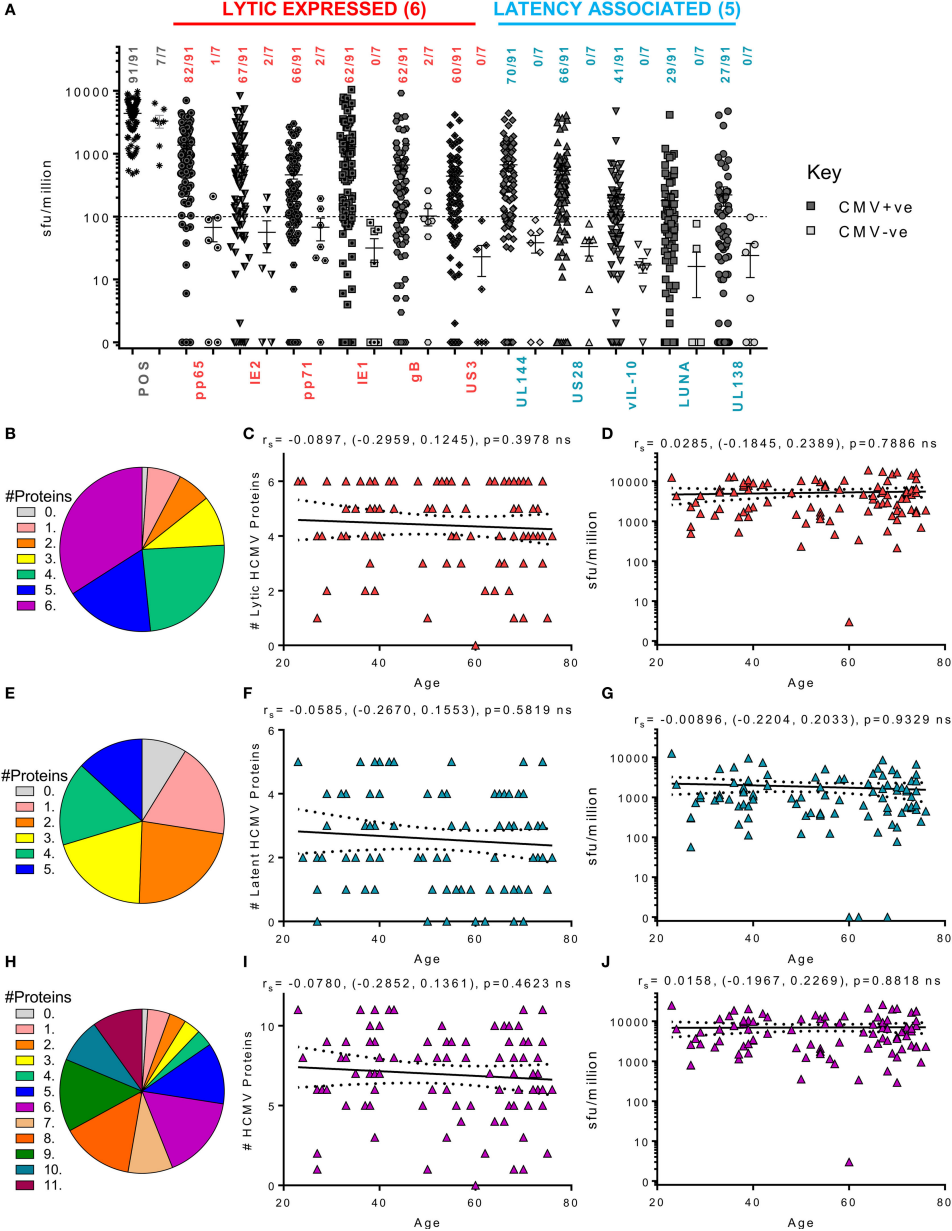
Magnitude and breadth of CD8^+^ T cell IFNγ response to human cytomegalovirus (HCMV) proteins. The IFNγ secreting CD8^+^ T cell response to 6 HCMV proteins only expressed during lytic infection: pp65, IE2, pp71, IE1, gB, US3, and 5 HCMV latency-associated proteins: UL144, US28, vIL-10, LUNA, and UL138 were measured in a cohort of 91 HCMV seropositive and 7 seronegative donors. The production of IFNγ was measured using an IFNγ FluoroSpot detection method; with the results converted to spot forming units/million cells (sfu/million) with background counts subtracted. The response to the lytic expressed proteins (red), latency associated (blue), and the positive control by all 98 donors are summarized **(A)** with HCMV seropositive donors (dark) and HCMV seronegative donors (light) both illustrated. The positive response threshold cutoff of 100 sfu/million is shown (dashed line) and the proportion of donors with a positive response to each HCMV protein is indicated. The proportion of the 91 seropositive donors producing a positive response to 1 or more of the 6 Lytic expressed proteins **(B)**, 5 latency-associated proteins **(E)** or all 11 HCMV proteins **(H)** are summarized as pie charts with the key to segments for each graph shown. Graphs illustrating the breadth of HCMV seropositive donors response to HCMV proteins correlated with age are illustrated for lytic expressed **(C)**, latency associated **(F)**, and all 11 proteins **(I)**; also shown is the summed IFNγ response to lytic **(D)**, latent **(G)**, and all proteins **(J)** correlated with age. Spearman rank correlation [Spearman *r*_s_ (95% confidence intervals) and *p* values] results are indicated on each graph.

Figure 3 summarizes the results from the screen of 98 donors for CD8^+^ IFNγ T cell responses. A majority of the HCMV seropositive donors analyzed had an above threshold (100 sfu/million) CD8^+^ IFNγ T cell response to the six lytic proteins analyzed as well as responses to the latency-associated proteins UL144 and US28 proteins (Figure 3A). We noted positive CD8^+^ T cell responses to LUNA (31.8% of donors) and UL138 (29.6% of donors), which while present in our previous study, using an enzymatic ELISPOT method, were below the positive response threshold (53) because this was a much less sensitive detection system. The frequency of individual donors who produced CD8^+^ T cell responses to 1 or more HCMV proteins is presented as pie charts for the lytic expressed proteins (Figure 3B), latency-associated proteins (Figure 3E) and for all HCMV proteins (Figure 3H). These analyses shows that a majority of the donors produced a response to 5 or 6 lytic proteins (51.6%—blue and deep pink segments Figure 3B), that 29.7% of the donor cohort responded to 4 or 5 of the latency-associated proteins (green and blue segments Figure 3E) and, overall, 47.2% of the cohort responded to 8 or more HCMV proteins (orange, dark green, teal, and purple segments, Figure 3H). The broad range of responses to lytic, latent and all HCMV proteins observed were also maintained with age (Figures 3C,F,I, respectively). An analysis of whether increasing age alters the magnitude of the CD8^+^ T cell IFNγ response to HCMV revealed no impact on the 11 individual proteins (data not shown) or the summed responses to lytic (Figure 3D), latent (Figure 3G), or all (Figure 3J) HCMV proteins examined.

**Figure 4:**
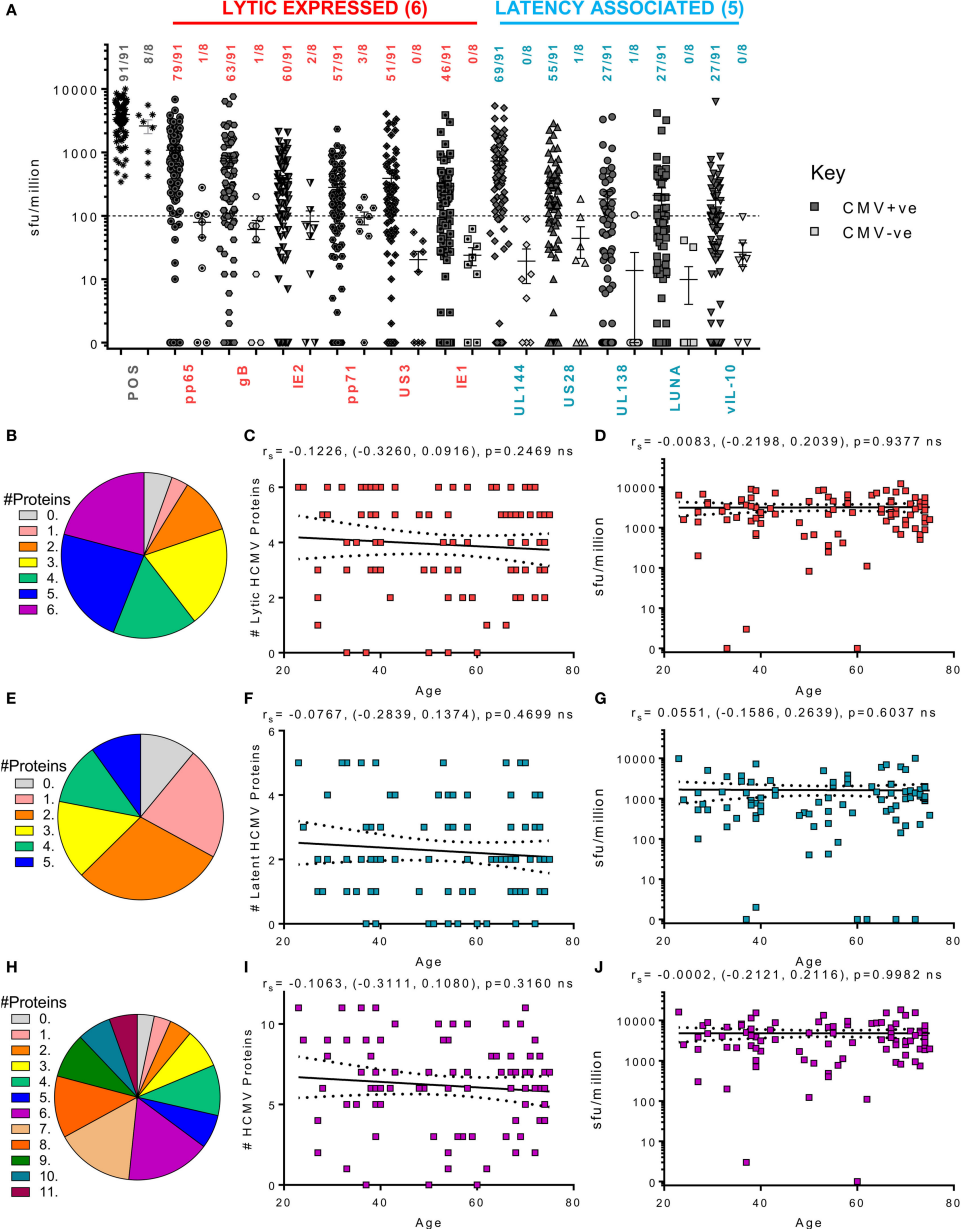
Magnitude and breadth of CD4^+^ T cell IFNγ response to human cytomegalovirus (HCMV) proteins. The IFNγ-secreting CD4^+^ T cell response to 6 HCMV proteins only expressed during lytic infection: pp65, IE2, pp71, IE1, gB, and US3 (red) and 5 HCMV latency-associated proteins: UL144, US28, vIL-10, LUNA, and UL138 (blue) were measured in a cohort of 91 HCMV seropositive and 8 seronegative donors. The production of IFNγ was measured using an IFNγ FluoroSpot method; with the results converted to spot forming units/million cells (sfu/million) with background counts then subtracted. The response to the HCMV proteins and the positive control by all 99 donors are summarized **(A)** with HCMV seropositive donors (dark) and HCMV seronegative donors (light) both illustrated. The positive response threshold cutoff of 100 sfu/million (dashed line) and the proportion of donors with an above threshold response to each HCMV protein is indicated. The proportion of the 91 seropositive donors producing a positive IFNγ response to 1 or more of the 6 Lytic expressed proteins **(B)**, 5 latency-associated proteins **(E)** or all 11 HCMV proteins **(H)** are summarized as pie charts with the key to segment color for each graph shown. Graphs illustrating the breadth of HCMV seropositive donors IFNγ response to HCMV proteins correlated with age are illustrated for lytic expressed **(C)**, latency associated **(F)**, and all 11 proteins **(I)**; also shown is the summed IFNγ response to lytic **(D)**, latent **(G)**, and all proteins **(J)** correlated with age. Spearman rank correlation [Spearman *r*_s_ (95% confidence intervals) and *p* values] results are indicated on each graph.

We also examined the CD4^+^ T cell responses of the donor cohort to the same 11 HCMV proteins in 99 donors. As observed for the CD8^+^ T cell responses, the majority of the HCMV seropositive donor cohort produced an above threshold IFNγ response to all the lytic expressed proteins but also latency-associated UL144 and US28 (Figure 4B). The responses to the lytic expressed proteins by CD4^+^ T cells have already been reported in a subset of this donor cohort (63); however, the observation that both UL144 and US28 proteins induce T cell responses in the majority of HCMV seropositive donors has not previously been reported. Only 29.6% of the donor cohort examined produced an above threshold CD4^+^ IFNγ response to UL138, LUNA, and vIL-10 latency-associated proteins; this is a similar frequency to that seen in the CD8^+^ T cell compartment and not dissimilar to the percentage of responding donors for UL138 and LUNA CD4^+^ T cell responses previously reported in a small-scale study (53). The ability of individual donors to mount CD4^+^ IFNγ responses to multiple HCMV proteins is summarized as pie charts (Figures 4B,E,H). In contrast to the CD8^+^ T cell IFNγ response routinely seen to 5 or 6 lytic proteins, fewer donors were capable of mounting responses to 5 or 6 of the lytic expressed HCMV proteins (43.9%—blue and deep pink segments, Figure 4B). This trend was maintained in response to the latent proteins (22% responding to 4 or 5 proteins—green and blue segments, Figure 4E) and, overall, only 33% of the donor cohort responded to 8 or more of the examined HCMV proteins (Figure 4H—orange, dark green, teal, and purple segments). Despite this lower proportion of HCMV seropositive donors responding to many HCMV proteins, the overall breadth of the CD4^+^ IFNγ T cell response remained stable with increasing donor age which shows that there was no significant increase or decrease in the number of proteins an individual responded to within the lytic (Figure 4C) or latent group of proteins (Figure 4F) or to all 11 proteins examined (Figure 4I). Also, we did not observe an effect of donor age on the magnitude of the response to the individual HCMV proteins (data not shown) or to the summed responses to the 6 lytic proteins (Figure 4D), 5 latent proteins (Figure 4G), or to the summed response of all 11 proteins (Figure 4J).

**Figure 5:**
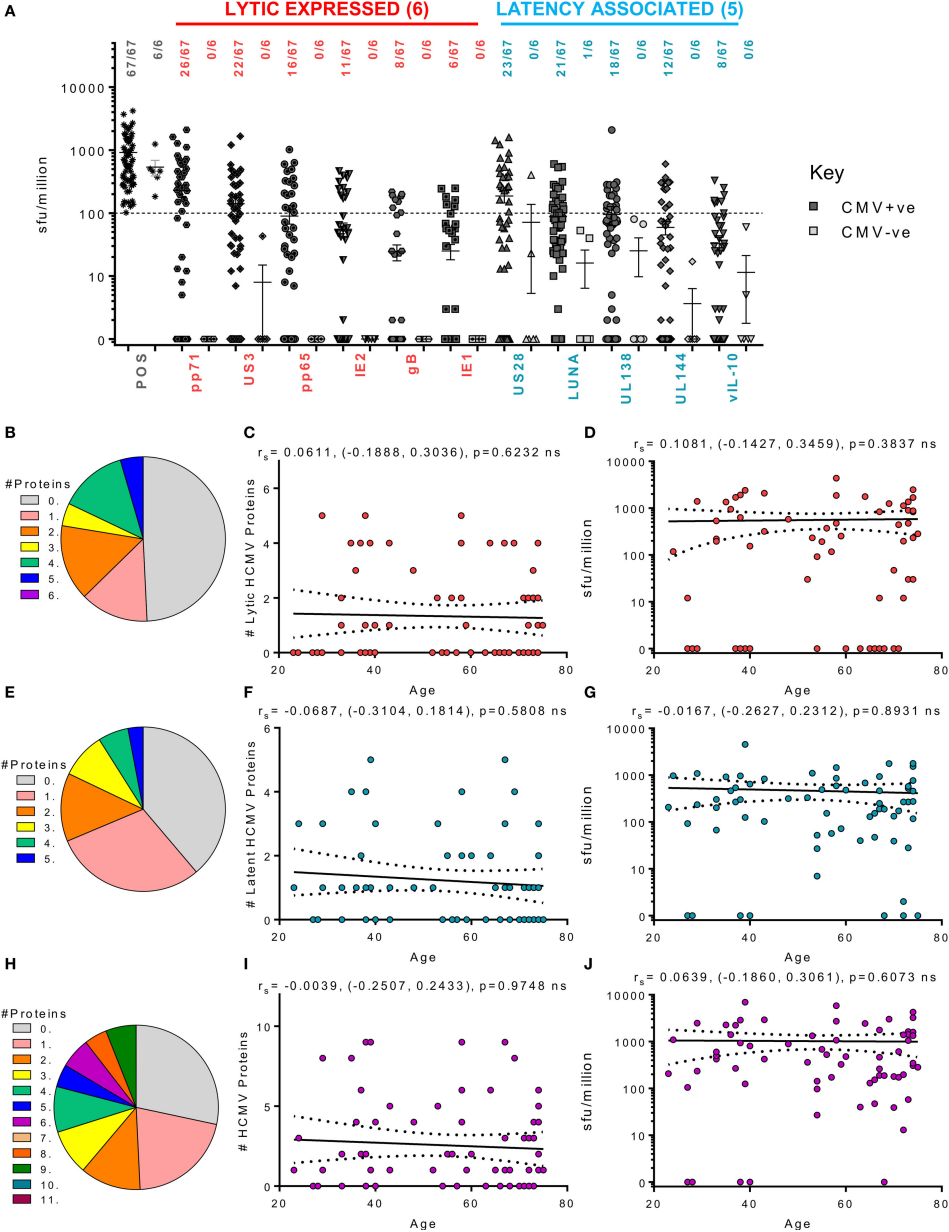
Magnitude and breadth of CD4^+^ T cell IL-10 response to human cytomegalovirus (HCMV) proteins. The IL-10-secreting CD4^+^ T cell response to 6 HCMV proteins only expressed during lytic infection: pp65, IE2, pp71, IE1, gB, and US3 (red) and 5 HCMV latency-associated proteins: UL144, US28, vIL-10, LUNA, and UL138 (blue) were measured in a cohort of 67 HCMV seropositive and 6 seronegative donors. The production of IL-10 was measured using an IL-10 FluoroSpot method; with the results converted to spot forming units/million cells (sfu/million) with background counts then subtracted. The response to the HCMV proteins and the positive control by all 73 donors are summarized **(A)** with HCMV seropositive donors (dark) and HCMV seronegative donors (light) both illustrated. The positive response threshold cutoff of 100 sfu/million (dashed line) and the proportion of donors responding to each HCMV protein is indicated. The proportion of the 67 seropositive donors producing a positive IL-10 response to 1 or more of the 6 lytic expressed proteins **(B)**, 5 latency-associated proteins **(E)** or all 11 HCMV proteins **(H)** are summarized as pie charts with the key to segment color for each graph shown. Graphs illustrating the breadth of HCMV seropositive donors IL-10 response to HCMV proteins correlated with age are illustrated for lytic expressed **(C)**, latency associated **(F)**, and all 11 proteins **(I)**; also shown is the summed IL-10 response to lytic **(D)**, latent **(G)**, and all proteins **(J)** correlated with age. Spearman rank correlation [Spearman *r*_s_ (95% confidence intervals) and *p* values] results are indicated on each graph.

We next examined the ability of CD4^+^ T cells to produce cIL-10 following stimulation with our 11 candidate HCMV proteins. Cellular IL-10 levels were measured in 73 HCMV donors from the cohort (these donors having passed the quality control thresholds outlined in the methods). Although we have already shown that lytically expressed proteins pp71 and US3 can induce cIL-10 production by CD4^+^ T cells in a small subset of this donor cohort (63), in this larger donor cohort, pp71 (38.8%), US3 (32.8%), and pp65 (23.8%) are the most common lytic proteins to trigger an above threshold cIL-10 CD4^+^ T cell response. The latency-associated proteins, US28 (34.3%), LUNA (31.3%), and UL138 (26.8%), also frequently induced a CD4^+^ specific cIL-10 response in this donor cohort. In contrast to the ability of donors to produce IFNγ T cell responses to multiple HCMV proteins, a positive cIL-10 response to any 1 of the 11 HCMV proteins examined was absent in 19 of 67 seropositive donors (gray segment—Figure 5H) and no donors produced responses to more than 9 of the 11 HCMV proteins. When examining the response to the 6 lytic proteins, about half of the 67 donors (49.3%) did not produce a cIL-10 response (gray segment—Figure 5B). Despite this more limited breadth of the response, 70% of the donors examined produced an above threshold cIL-10 response to 1 or more HCMV protein. The ability of an individual donor to produce a cIL-10 response to HCMV proteins was not affected by age (Figures 5C,F,I) and neither was the magnitude of the responses to each of the 11 HCMV proteins (data not shown). The relationship of the total cIL-10 responses, for each donor, to the 6 lytic proteins (Figure 5D), 5 latent proteins (Figure 5G), and all 11 proteins (Figure 5J) was also stable with donor age. Overall, the data presented show that the breadth and magnitude of the IFNγ and cIL-10 HCMV-specific T cell responses, within this donor cohort, do not show any impact of either increasing donor age or putative long-term carriage of the virus on these HCMV-specific T cell responses.

### CD4^+^ T Cells Specific for LUNA, UL138, pp71, US3, and US28 Proteins Are More Frequently Biased toward Expression of cIL-10 than IFN γ and This Was Not Affected by Donor Age

**Figure 6:**
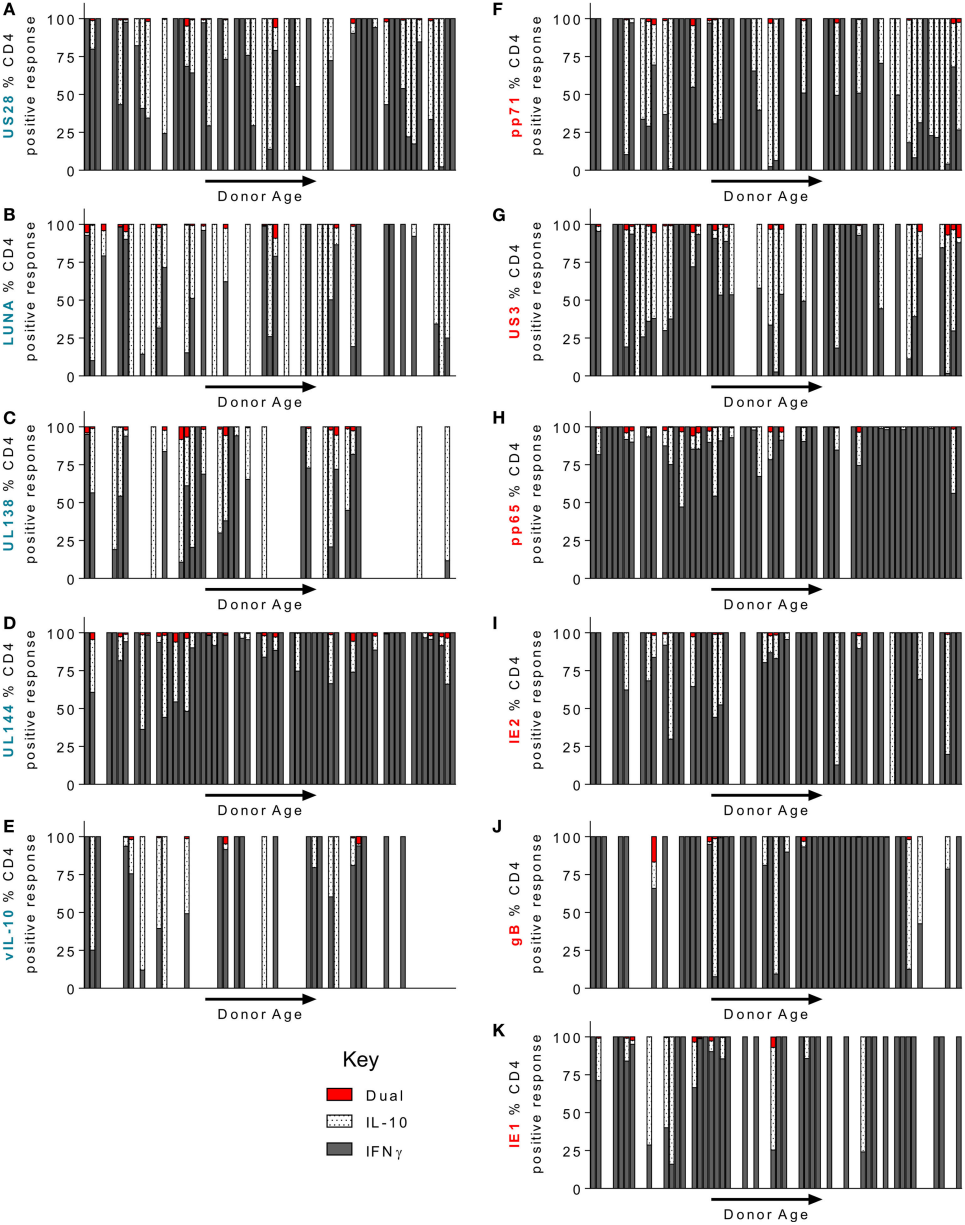
CD4^+^ T cell donor responses to human cytomegalovirus (HCMV) LUNA, UL138, pp71, US3, and US28 proteins were more frequently IL-10 biased. The frequency of CD4^+^ T cells that secrete IFNγ or IL-10 or both in response to stimulation by HCMV proteins was measured simultaneously using a dual IFNγ/IL-10 FluoroSpot assay. 67 HCMV seropositive donors were analyzed, only donors with above threshold responses for either IFNγ or IL-10 (100 sfu/million) to each protein are shown. The IFNγ (dark gray), IL-10 (white spotted), and dual cytokine (red) responses of the donor cohort to US28 **(A)**, LUNA **(B)**, UL138 **(C)**, UL144 **(D)**, vIL-10 **(E)**, pp71 **(F)** US3 **(G)**, pp65 **(H)**, IE2 **(I)**, gB **(J)**, and IE1 **(K)** are shown as a percentage of the total CD4^+^ T cell (IFNγ^+^ IL-10) response of each donor, the donors are arranged along the *x*-axis in increasing age order. The lytic expressed proteins axis label is in red **(F–K)** and the latency-associated protein responses are labeled in blue **(A–E)**.

Using the FluoroSpot technology, we were able to ask whether CD4^+^ T cell responses to our candidate, HCMV proteins was dominated by either IFNγ or IL-10 secretion or whether it was comprised of cells that secrete both cytokines. Figure 6 shows the relative cytokine composition of the CD4^+^ T cell response to each of the 11 HCMV proteins examined for donors who generated an above threshold response (>100 sfu/million) for either cytokine. Overall, we found that IFNγ and cIL-10 are generally produced by distinct populations of CD4^+^ T cells, as dual secretors were very rare (red bars—Figure 6). The CD4^+^ T cell responses to UL144 (Figure 6D), gB (Figure 6J), pp65 (Figure 6H), IE1 (Figure 6K), and IE2 (Figure 6I) proteins were dominated by IFNγ secretion. In contrast, the donor cohort responses to the proteins UL138 (Figure 6C), LUNA (Figure 6B), US28 (Figure 6A), vIL-10 (Figure 6E), pp71 (Figure 6F), and US3 (Figure 6G) showed more cIL-10 secretors (white spotted bars). Although there was no significant change in the magnitude of the CD4^+^ T cell IL-10 response to HCMV proteins with age (summarized Figure 5), we were interested to see if there was a change in the proportion of IFNγ and IL-10 secretion by CD4^+^ T cells within individuals during long-term viral carriage. The data presented in Figure 6 are arranged with donor age along the *x*-axis and does not show any obvious changes in the composition of the positive CD4^+^ T cell response. Analysis of the proportion of donors in which the majority of the CD4^+^ T cell responses was secretion of cIL-10 (i.e., greater than 50% of the total CD4^+^ T cell response of the individual to each HCMV protein) revealed that for LUNA 48.5% of responding donors had a dominant cIL-10 response (Figure S3A in Supplementary Material). UL138, pp71, US3, and US28 also elicited a greater than 50% IL-10 response in more than a third of the donor cohort (42.8, 38.4, 34, and 33.3%, respectively; Figure S3A in Supplementary Material). When looking at the breadth of the cIL-10 dominant responses with donor age, there was no significant increase in the breadth of HCMV proteins and individual produced a majority cIL-10 response toward for all proteins (Figure S3B in Supplementary Material), lytic proteins (Figure S3C in Supplementary Material), or latent-associated proteins (Figure S3D in Supplementary Material).

### The Magnitude of Latent HCMV DNA Load in CD14^+^ Monocytes Is Not Affected by Donor Age in the ARIA Cohort

**Figure 7:**
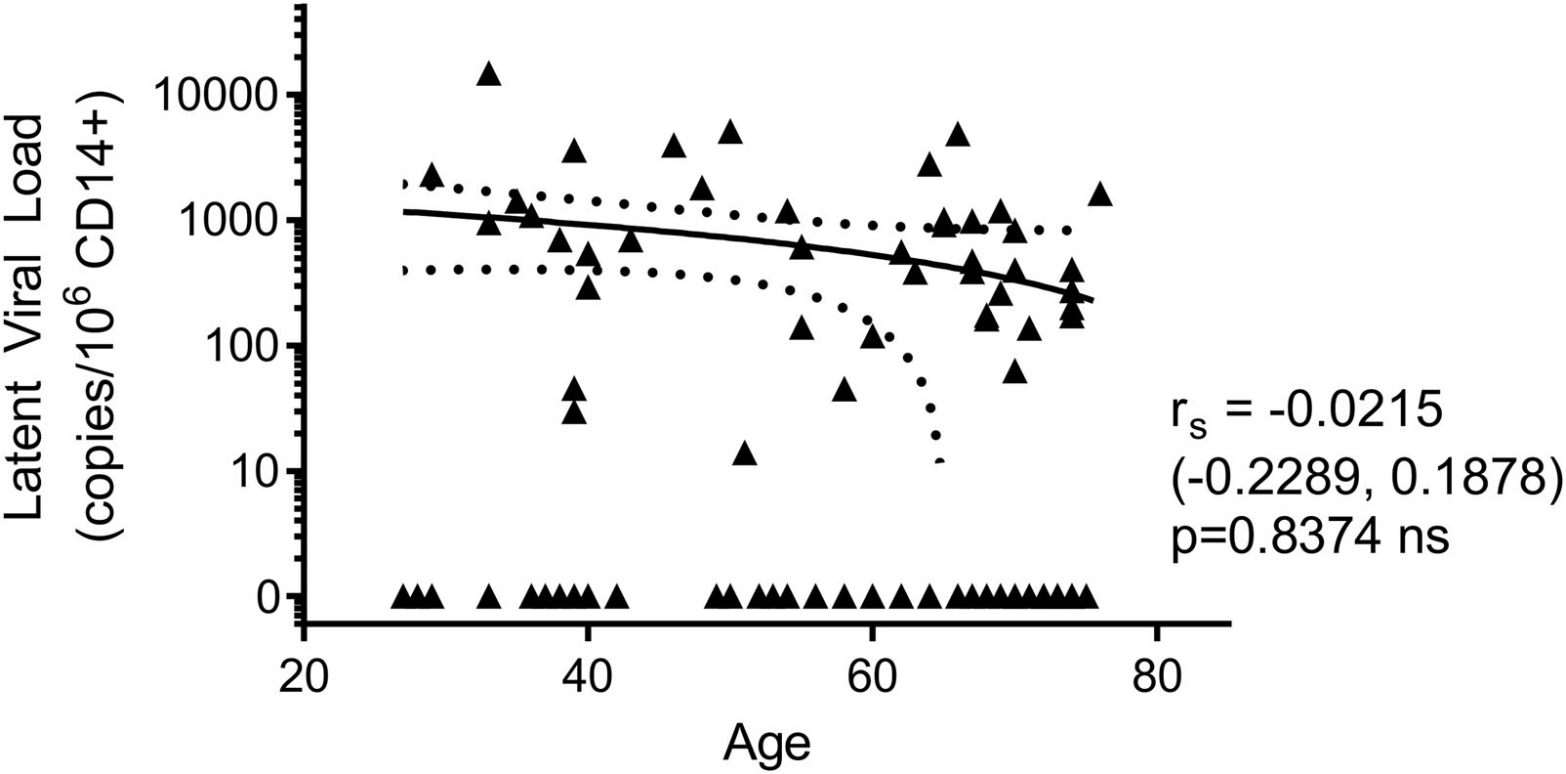
There was no effect of donor age on the magnitude of latent human cytomegalovirus (HCMV) load in CD14^+^ monocytes. The DNA of purified CD14^+^ monocytes was extracted and HCMV viral load detected using droplet digital PCR analysis. No HCMV was detected in 14 HCMV seronegative donors tested. The HCMV viral load (copies/10^6^ CD14^+^ cells) results from 94 HCMV seropositive donors are shown correlated with donor age. Spearman rank correlation [Spearman *r*_s_ (95% confidence intervals) and *p* values] analysis is indicated on the graph.

In addition to assessing the effect of increasing age on the T cell response to HCMV lytic and latent expressed proteins, the other principle aim of this study was to determine if there was an age-related effect on latent viral load. Consequently, we screened whole blood of all donors in the study for the presence of HCMV DNA using a quantitative real-time PCR assay. No viral DNA was detectable in the 14 HCMV seronegative donors and of the 105 HCMV seropositive donors, viral genome was only detected in 1 of these (274 copies/ml whole blood). The donor with detectable HCMV in whole blood also had an inverted CD4:CD8 ratio and above average numbers of differentiated memory CD8^+^ T cells, data summarized in Figure S4 in Supplementary Material. During latent HCMV infection, virus is known to reside in CD34^+^ hematopoietic stem cells and derivative CD14^+^ monocytes (64). Using a sensitive ddPCR approach (54), we quantified the number of copies of HCMV present in isolated CD14^+^ monocytes from all donors. In total, we assessed 108 HCMV seropositives and negatives for HCMV DNA present in CD14^+^ cells; of these, no copies of viral genome were detected in the 14 HCMV seronegative donors. We did, however, detect HCMV genomes in 43 of 94 (45.7%) of CD14^+^ monocytes from HCMV seropositive donors (51 of 94 were below the level of detection of this assay, 1 genome in 60,000 cells); the latent viral load (copies HCMV/million CD14^+^ cells) for the 94 seropositive donors, relative to donor age, is summarized in Figure 7. Within this ARIA donor cohort, we did not observe a significant relationship between age and the magnitude of the latent viral load.

### High Latent Viral Loads in CD14^+^ Monocytes Were Associated with both Increased Breadth and Frequency of IFNγ-Secreting HCMV-Specific T Cells

**Figure 8:**
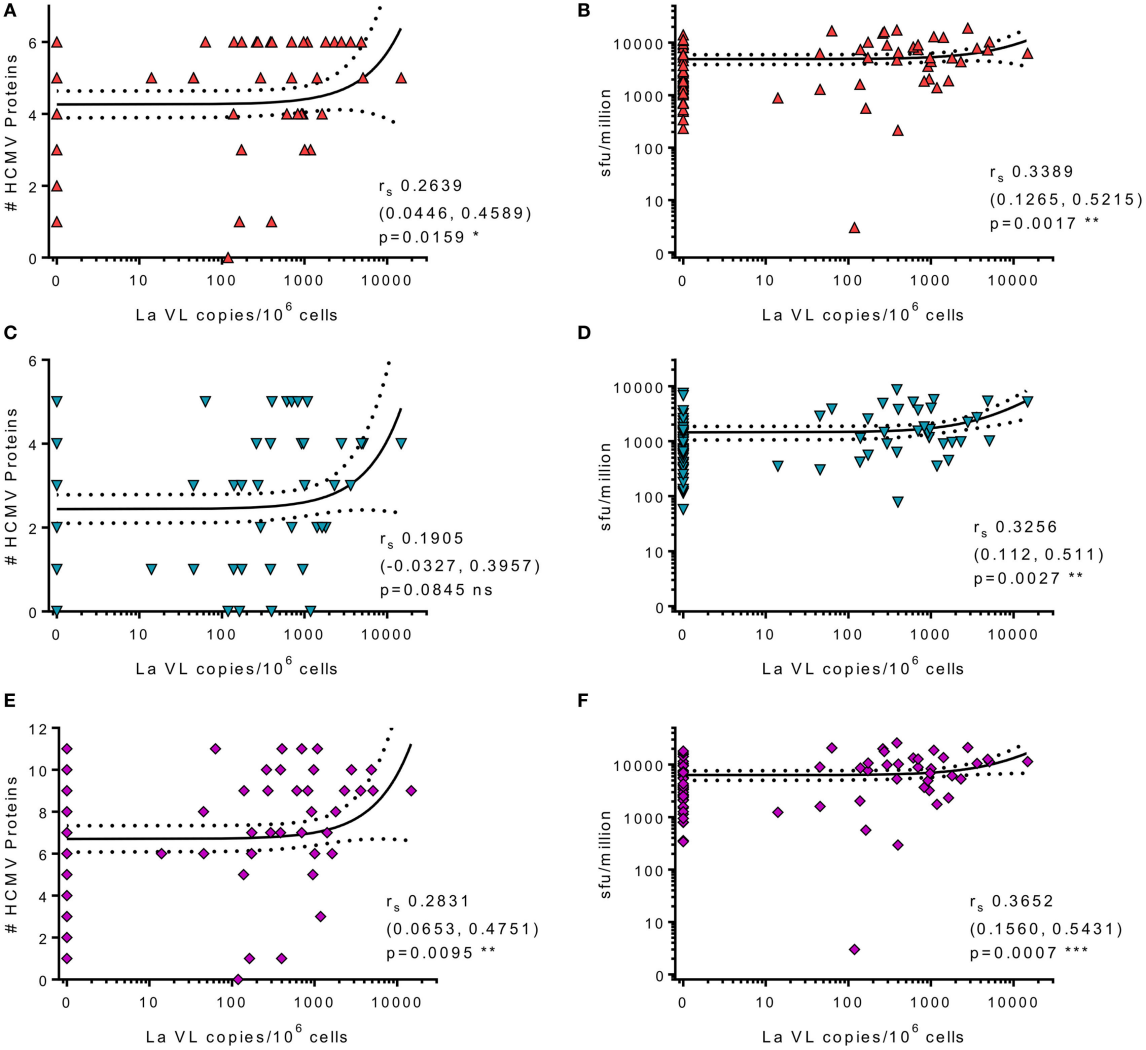
High levels of latent human cytomegalovirus (HCMV) load in CD14^+^ monocytes correlates with increased frequency and breadth of HCMV-specific IFNγ CD8^+^ T cell responses. The HCMV viral load (copies/10^6^ CD14^+^ cells) from 83 HCMV seropositive donors was correlated with CD8^+^ HCMV-specific T cell responses. Graphs illustrating the breadth (positive response) of individual donors CD8^+^ IFNγ response to the 6 lytic expressed (red) **(A)**, 5 latency-associated (blue) **(C)**, and all 11 HCMV proteins (purple) **(E)** correlated with CD14^+^ cells HCMV viral load are shown. The magnitude of the CD8^+^ IFNγ response summed for all protein groups is correlated with HCMV viral load for lytic (red) **(B)**, latent (blue) **(D)**, and all proteins (purple) **(F)**. Spearman rank correlation [Spearman *r*_s_ (95% confidence intervals) and *p* values] results are indicated on each graph.

Human cytomegalovirus is latently carried in CD34^+^ hematopoietic progenitor cells and subsequently in the periphery by monocyte derivatives from these cells (65). Virus reactivation from these myeloid lineage cells would activate HCMV-specific T cells and could drive increased frequencies, as well as potentially seeding more cells in the latent reservoir. Theoretically, increased frequency of latently infected cells could result in increased virus reactivation events, potentially resulting in induction of more T cell stimulation and, possibly, an increase in HCMV-specific antibody levels during lifelong persistence. Consequently, we assessed whether there was an association between HCMV-specific IgG levels and latent viral load, but these measures were unrelated (data not shown). We then assessed whether there was an association between the latent viral load and the CD8^+^ and CD4^+^ T cell responses to the individual HCMV proteins as well as to the magnitude and breadth of the total responses of each donor. We did not observe an association between latent load and the cIL-10 CD4^+^ response and there was only a significant association between the magnitude and breadth of the CD4^+^ IFNγ response to the subset of 6 lytic proteins and increased latent viral load (data not shown). There was a significant association with the summed total of the CD8^+^ T cell response to lytic (Figure 8B), latent (Figure 8D), and all proteins (Figure 8F). Also, high viral copy latent load correlated significantly to the breadth of the CD8^+^ T cell responses to lytic (Figure 8A) and all HCMV proteins (Figure 8E), but not to the latent proteins only (Figure 8C).

## Discussion

The aims of this study were to determine whether HCMV-specific CD4^+^ T cells secreting cIL-10 increase with age and long-term viral carriage and to determine whether there are changes in breadth and frequency of the IFNγ-secreting T cell response to HCMV infection in healthy older donors. We also wanted to measure the latent viral load of HCMV DNA in a large donor cohort for the first time and assess whether donors aged over 65 years manifested changes in immune cell numbers indicative of immunosenescence. Using an age cross-sectional study methodology, we recruited a donor cohort spanning six decades (23–78 years) and measured virological and immunological parameters. The donors were recruited by the Cambridge Bioresource from their Biobank of volunteers who live predominantly in areas local to Cambridge and the East Anglian Region of the UK. Donors were recruited based on HCMV serostatus and by excluding donors suffering from immune altering illnesses or under treatment for these conditions, such that all participants could be safely considered to be generally healthy.

We analyzed the CD4^+^ and CD8^+^ T cell compartments in peripheral blood and observed a loss of naïve CD4^+^ and CD8^+^ T cell numbers as well as a corresponding loss of total CD4^+^ and CD8^+^ T cell numbers with increasing age. The age-related loss of naïve T cells numbers is a well-established phenomenon due to the involution of the thymus and decreased T cell output (66) and has been observed in most studies of aging populations (24). In our study, there was no accumulation of memory T cell populations (measured in absolute numbers) within this cohort, which has also been observed in other studies when using absolute numbers (52, 67). However, when expressed as a percentage of the CD8^+^ T cell compartment, there was a significant age-related accumulation of differentiated T_EMRA_ (CD27^−^CD45RA^+^) and Late stage (CD27^−^CD28^−^) memory cell populations as has been previously reported (24). It is likely that the increase in percentage (relative frequency) of differentiated memory T cell populations previously reported in aged cohorts was due to the decrease in the absolute size of the overall CD8^+^ T cell compartment, which results in an increase in the proportion of memory cells even if the absolute numbers do not increase (1, 52, 67).

Previous investigations into the impact of HCMV persistence on immunosenescence in older people have reported a range of immune parameters and HCMV-specific markers altering with age. These include the IRP, defined by a collection of markers which, taken together, were suggested to be indicative of increased mortality in the elderly and which included an inversion of the CD4:CD8 ratio, expansion of CD8^+^ CD28^null^ and CD8^+^ T_EMRA_ memory T cells and HCMV seropositivity (8, 9, 12, 13). There have also been reports of HCMV-specific IgG levels increasing in older donors (54, 68, 69) as well as accumulation of HCMV-specific T cells with age [summarized in Ref. (24)]. Similarly, it has been suggested that there is an age-related increase in levels of HCMV DNA in blood (37), urine (36), and an increase in latent viral genome copy number in CD14^+^ cells of donors aged over 70 years (54). Overall, as our donor cohort exhibited a normal aging immune phenotype, we examined the impact of HCMV seropositivity on T cell memory phenotype within the study group. There were no significant differences in naïve T cell numbers between aged HCMV seropositive compared to aged HCMV seronegative donors in our cohort and we only observed an inverted CD4:CD8 ratio in 10% of the seropositive donor cohort; donors exhibiting this phenotype were distributed throughout the age categories. We did see an increase in the numbers of differentiated T cells in HCMV seropositive donors of all ages compared to seronegatives, confirming that our study participants have a similar T cell phenotype to that observed in many previous studies of HCMV infection (24). There was, however, no association between increasing donor age and higher levels of HCMV IgG nor was there an increase in the breadth and frequency of the HCMV-specific T cell IFNγ response or CD4^+^ cIL-10 response to the 11 HCMV proteins examined within the study group. We also did not detect increased copies of latent HCMV genome in CD14^+^ monocytes of our older donors. The separate impact of HCMV infection from aging on the differentiation of T cells has been observed in other population studies (19, 21) and the kidney transplant primary infection model and reports from primary infection has shown a rapid acquisition of a more differentiated T cell phenotype in the months following initial infection (30, 70–72). Furthermore, we observed a significant association between high latent viral loads and higher frequency HCMV-specific CD8^+^ T cell responses, which was again irrespective of donor age. These observations alongside the increased numbers of differentiated memory T cells suggest that, within this healthy donor cohort, it is HCMV infection, rather than the age of the donor, which leads to increased differentiation of the T cell population and expansion of HCMV-specific T cells.

Work on donor cohorts from different geographical locations have reported different findings from the original Swedish studies which described the IRP (8, 9, 12, 13), these have included a lack of “inflation” of HCMV-specific T cells with age despite high HCMV seroprevalence in the aged donor groups (73) and the association of a naïve T cell phenotype in HCMV seropositive old people with increased morbidity in Belgium (74). HCMV seroprevalence varies depending on geographical location and socio-economic status (6, 75); in the developed world between 30 and 70% of populations are HCMV seropositive, with acquisition of the virus increasing with age (76). In contrast, in developing countries, seroprevalence can be higher than 90% with acquisition of the virus commonly occurring in early childhood (30, 76). Consequently, the disparate observations reported as consequences of HCMV infection in different aged donor cohorts may be a result of geography as well as other biological parameters such as exposure to infectious diseases, vaccination history, and the current health of the participants. It has also been shown in other studies of very old cohorts that increased HCMV IgG levels and differentiated CD4^+^ T cells are associated with elderly individuals in poor health (27), and there are also a number of studies associating HCMV seropositivity and higher HCMV IgG titers with poor outcomes from cardiovascular disease (14, 15, 17, 18). Our view is that, in some cohorts that have been studied, aged donors suffering from, e.g., heart disease, cancer or neurodegenerative disorders may not control virus efficiently leading to increased HCMV IgG levels or HCMV DNAemia and concomitant increased numbers of differentiated memory T cell populations and an inverted CD4:CD8 ratio, thereby confounding some studies.

One of our aims was to address the production of cIL-10 by HCMV-specific CD4^+^ T cells within a large donor cohort in order to assess how prevalent the production of this suppressive cytokine is by HCMV antigen-specific T cells and whether this response increases in older donors. Evidence from mouse models of MCMV infection have shown that production of cIL-10 can result in reduced viral clearance and a reduction in production of IFNγ by MCMV-specific T cells (49, 50). This could provide an explanation for the observation that, despite a functional immune response preventing overt HCMV mediated disease, older donors have detectable HCMV DNA in blood and urine (36, 37). In some HCMV studies, increases in inducible regulatory CD4^+^ T cells have been reported in older people with this being associated with vascular pathology in these individuals (47). Similarly, it has also been suggested that the HCMV-specific CD4^+^ CD28^−^CD27^−^T cell population, reported as expanded in HCMV seropositive older people (77), contains a T regulatory population characterized by FoxP3 and CD25^hi^ expression (45). As already discussed, there was no accumulation of the cIL-10 CD4^+^ T cell response with increasing donor age in this cohort; we were also interested to see if there was a shift in the bias of the responding CD4^+^ T cells to individual HCMV proteins from IFNγ to IL-10 or *vice versa*. The results confirmed our previous observation that the production of cIL-10 by CD4^+^ T cells is more likely to be in response to latency-associated proteins (53); in this cohort, almost 50 and 40% of donors produced a majority cIL-10 response to stimulation by the LUNA and UL138 peptide pools, respectively, regardless of donor age. Similarly, other latency-associated proteins included in this study, US28 and vIL-10, also showed a number of donors biased toward cIL-10 production, which is in contrast to the response toward many of the lytically expressed proteins included in this study.

The use of the ddPCR protocol (54) has enabled better quantification of the levels of latent HCMV genomes in the CD14^+^ cell compartment. We were able to detect and quantify latent HCMV genomes in 45.7% of examined HCMV seropositive donors comparing favorably to the 36% detection rate in HCMV positive donors described recently by ddPCR (54). Our ability to quantify latent HCMV load in our donor cohort led to a particularly interesting observation with respect to HCMV-specific T cell response. As already noted, high copy numbers of latent HCMV detected in CD14^+^ monocytes significantly correlated with an increase in the breadth and magnitude of the HCMV-specific CD8^+^ T cell response measured by IFNγ secretion. From this result, we hypothesize that higher viral genome copy number was a result of an accumulation of reactivation events over the time, resulting in viral replication and reseeding of the latent CD34^+^ cellular pool; consequently, this production of viral proteins stimulates and activates HCMV-specific memory T cell response leading to an increase in frequency of these cells. The virus most likely employs its immune evasion functions to create a window of opportunity to allow reactivation from latency and the production of new virions despite the presence of a primed antiviral immune response (5). In older donors, uncontrolled reactivation of HCMV subsequently causing either disease or other medical complications has not been observed, and HCMV DNA has not been routinely detected in the blood (36, 78), apart from in a Japanese cohort study, but the DNA positive detection rate was only 4.3% of donors aged 60–69 years (37). However, there is evidence that older people may not control virus replication as adequately as the young, as HCMV DNA has been detected in other bodily fluids in the old (36). Within this study, our exclusion criteria may have precluded recruitment of donors who had less effective control of virus replication resulting in low-level virus dissemination. In support of this conclusion, it is interesting to note that a single aged male donor with detectable HCMV DNA in whole blood did have an inverted CD4:CD8 ratio as well as an above average number of highly differentiated memory CD8^+^ T cell populations; they also had limited HCMV-specific T cell responses to our 11 candidate HCMV proteins (Figure S4 in Supplementary Material).

We have demonstrated that, in an East Anglian-based donor cohort which has a typical healthy aging profile, older HCMV seropositive donors do not exhibit the hallmark features of the IRP, differences in the breadth, and magnitude of their HCMV-specific IFNγ production, or that latent viral load was affected by age. Importantly, though we did see a significant relationship between high latent viral load and increased breadth and magnitude of the functional HCMV-specific CD8^+^ T cell responses, latent viral load did not correlate with increased numbers of differentiated memory T cell populations or HCMV-specific IgG. This, we believe, reflects the importance of including measurement of viral load in studies on the impact of HCMV infection in older donors as opposed to inferring the impact of the virus from measuring a variety of other immune parameters as has previously occurred. In a previous study in a Birmingham based old aged cohort, the authors observed an increase in HCMV-specific T cell responses alongside, an increase in latent viral carriage in donors aged over 70 years (54). While the authors do not present data correlating latent viral load with the frequency of HCMV-specific T cells, we think it possible in light of our findings, that in this older cohort study, the increase in HCMV-specific T cell responses in older donors could be associated with increased latent viral carriage.

Detection of low-level HCMV viremia in the blood of the old would be a strong indicator of a diminution of immune control; however, the results from our study group and others (36, 78) suggests this is rarely observed, probably because it would represent a significant loss of control. However, the presence of virus in other bodily fluids, e.g., saliva or urine could also indicate loss of immune control. It should be considered that chronic low-level persistent HCMV replication and an associated inflammatory environment could be important in particular old patients groups; there is epidemiological evidence that HCMV comorbidity plays a role in exacerbating cardiovascular disease (14, 15, 17, 18) and also with increasing impaired physical function and ill health (27, 29, 74, 79). Future investigations into the impact of HCMV infection in older people should also monitor latent viral carriage of the virus alongside measuring whether low-level viremia is present in the blood and other bodily fluids, e.g., urine or saliva; in order to improve our understanding of the impact of HCMV infection in the elderly.

## Ethics Statement

Ethical approval was obtained from University of Cambridge Human Biology Research Ethics Committee. Informed written consent was obtained from all donors in accordance with the Declaration of Helsinki (HBREC.2014.07).

## Author Contributions

SJ, MW, EP, and JS designed the project and experiments. SJ, GS, GO, and EP carried out the experiments. SJ and MW wrote the manuscript. SJ carried out statistical analysis and prepared figures. SJ and MW submitted this paper. All authors reviewed the manuscript.

## Conflict of Interest Statement

The authors declare that the research was conducted in the absence of any commercial or financial relationships that could be construed as a potential conflict of interest.
