# Supplementary material for: Latent Cytomegalovirus (CMV) Infection Does Not Detrimentally Alter T Cell Responses in the Healthy Old, But Increased Latent CMV Carriage Is Related to Expanded CMV-Specific T Cells
Source: Front Immunol. 2017 Jun 26;8:733. doi: 10.3389/fimmu.2017.00733 (PMC5483450; doi:10.3389/fimmu.2017.00733)
Supplement: Supplementary file 1 [file presentation_1.pdf]

Supplementary Figure 1 – Absolute Count Representative Analysis Gating Strategy

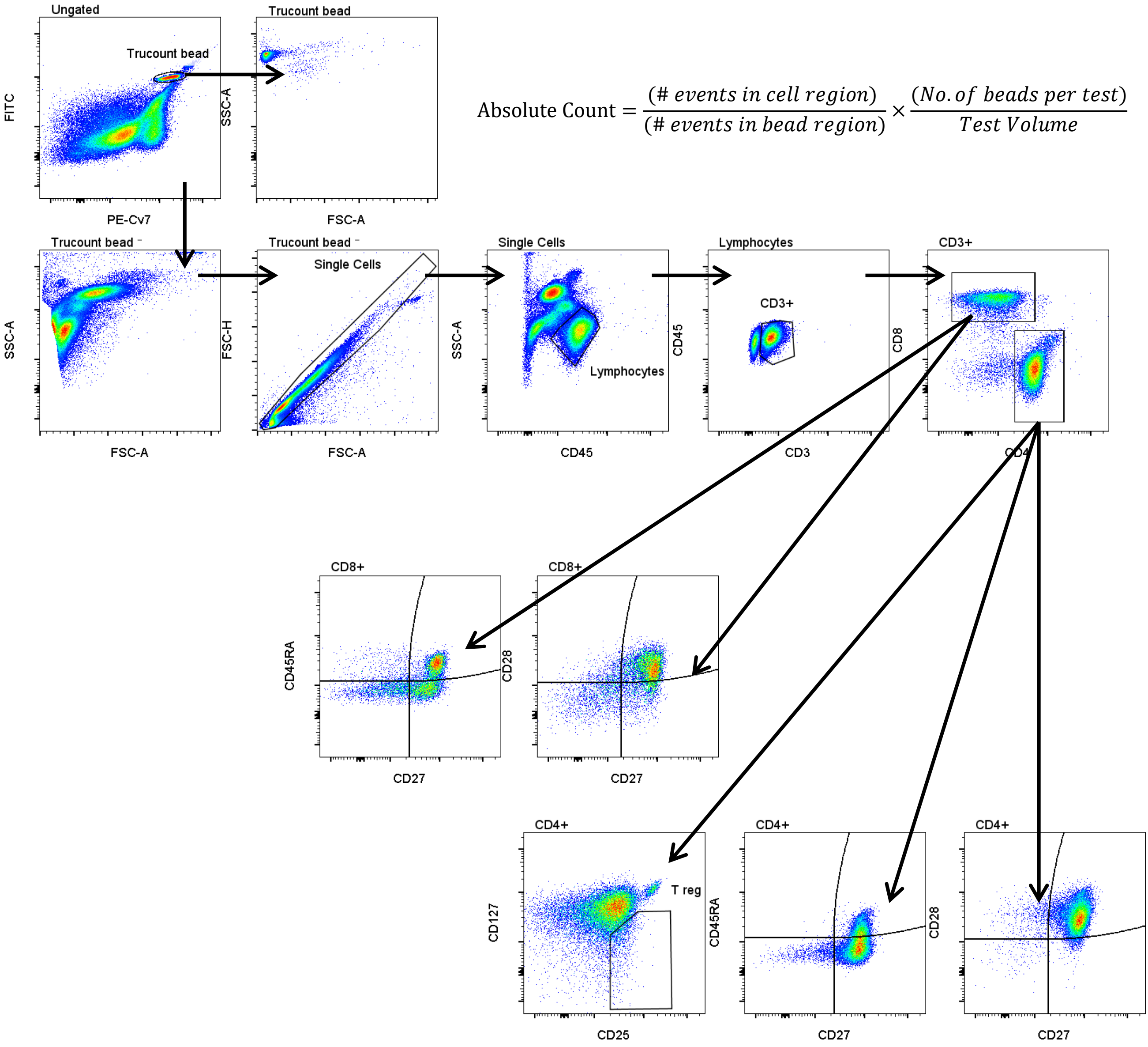

$$\text{Absolute Count} = \frac{(\#\ events\ in\ cell\ region)}{(\#\ events\ in\ bead\ region)} \times \frac{(No.of\ beads\ per\ test)}{Test\ Volume}$$

Supplementary Figure 1 – Absolute Count Representative Gating and population analysis.

Representative dot plots from 1 donor are shown illustrating the gating strategy for generating the absolute count data. First the trucount bead population was identified and then the trucount bead negative population (i.e. cells) were analysed by gating for single cells, then CD45^{hi} lymphocytes, CD3+ T cells, CD4+ and CD8+ expressing cells. The CD4+ and CD8+ T cell populations were further subdivided into 4 memory populations defined by expression of CD27 and CD45RA, and 4 differentiation populations defined by expression of CD27 and CD28 were identified and in CD4+ T cells a T_{reg} population defined as CD25^{hi} and CD127^{lo} were identified, all gate and quadrant positions were identified using the FMO controls. The formula used to calculate the absolute cell counts from the event numbers in each gate or quadrant is illustrated.

Supplementary Figure 2 – Impact of Ageing on T cell numbers CD4+ and CD8+ T cell Memory and Differentiation subsets

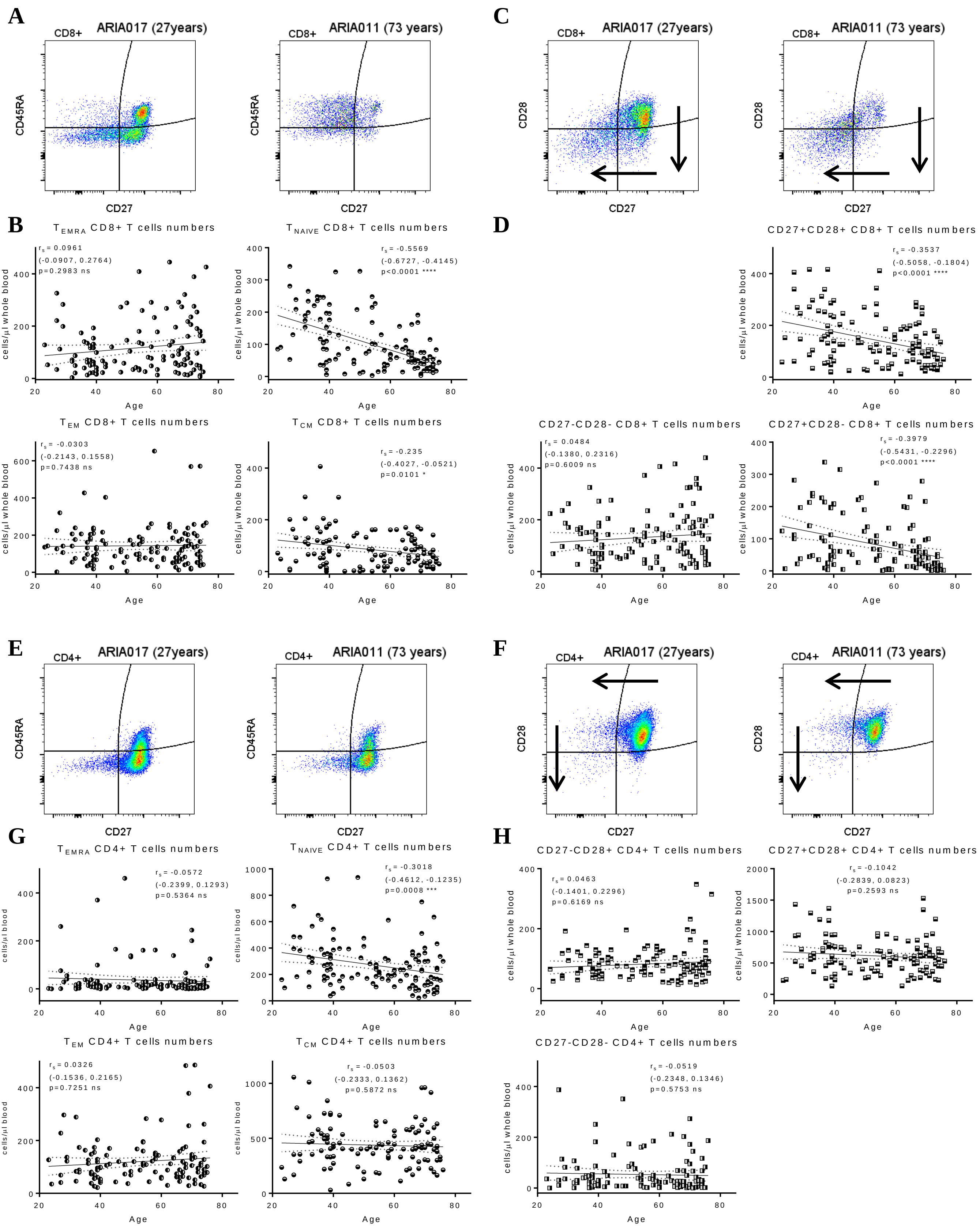

Supplementary Figure 2 – Impact of Ageing on CD4+ and CD8+ T cell memory and differentiation subsets numbers.

EDTA treated whole blood was stained and CD4+ and CD8+ T cells and subsets were enumerated. Representative dot plots from a young and an old HCMV sero-positive donor are illustrated showing the CD8+ memory (CD27 and CD45RA defined) (A) and differentiation (CD27 and CD28 defined) (C) and the CD4+ memory (E) and differentiation (F) phenotype; arrows on the CD8+ (C) and CD4+ (F) differentiation dot plots indicate the different pattern of CD27 and CD28 loss each T cell subset takes. Graphs illustrating the numbers of the four memory CD8+ T cell populations (B), three differentiation CD8+ T cell populations (D), four memory CD4+ T cell populations (G) and three differentiation CD4+ T cell populations (H) of the entire ARIA cohort (n=119) correlated to donor age are shown. The relationship of T cell subset numbers with donor age was analysed using Spearman rank correlation with the results indicated on each graph (r_s (95% Confidence Interval) and p value). The four memory populations defined by CD27 and CD45RA expression are: CD27+CD45RA+ (Naïve – T_{NAIVE}), CD27+CD45RA- (Central Memory – T_{CM}), CD27-CD45RA- (Effector Memory – T_{EM}) and CD27-CD45RA+ (Effector Memory CD45RA re-expressing cells – T_{EMRA}).

Supplementary Figure 3 – CD4+ T cell Donor responses to HCMV LUNA, UL138, pp71, US3 and US28 proteins more frequently express IL-10 instead of IFNγ. There is no impact of age on the dominance of the IL-10 response

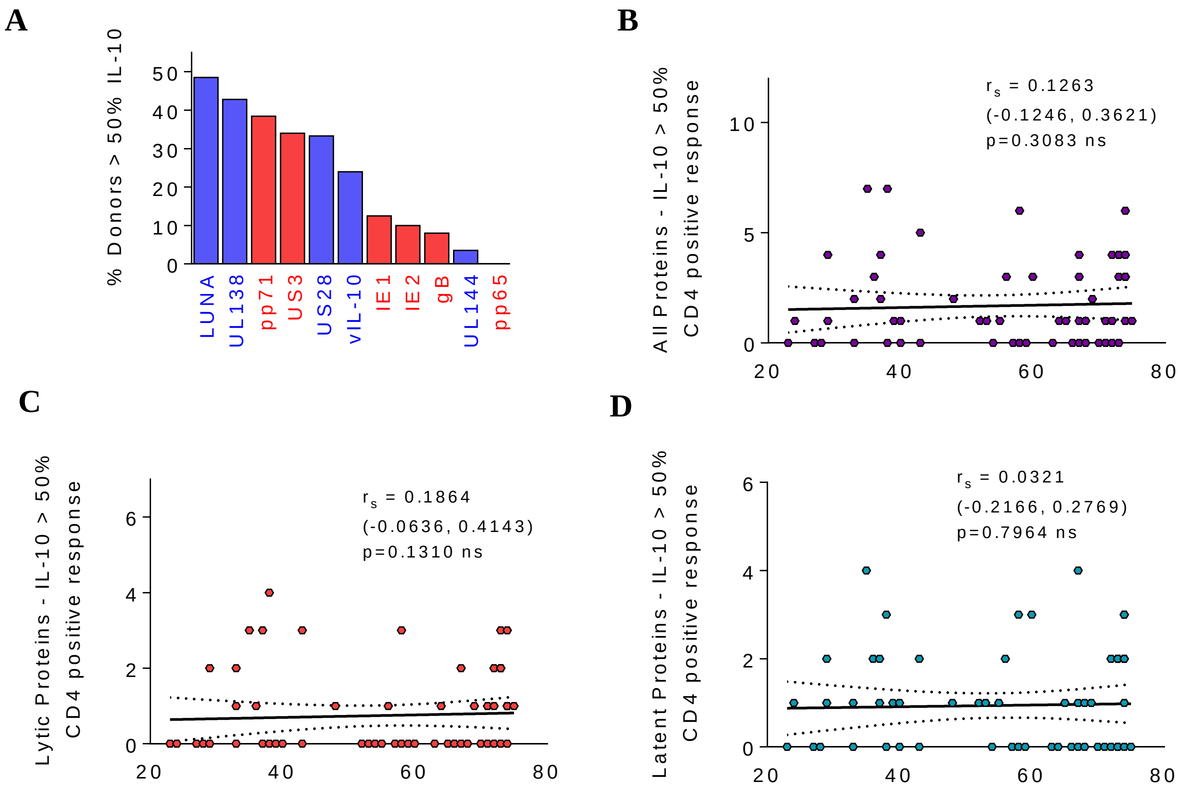

Supplementary Figure 3 – CD4+ T cell Donor responses to HCMV LUNA, UL138, pp71, US3 and US28 proteins more frequently express IL-10 instead of IFNγ. There is no impact of age on the dominance of the IL-10 response.

The number of donors whose positive CD4+ T cell response to each HCMV protein comprised of greater than 50% IL-10 production was enumerated from results presented in Figures 4, 5 and 6. The percentage of donors with an IL-10 response > 50% for each HCMV protein are shown and ranked (A). The number of proteins an individual produces a majority IL-10 CD4+ T cell response to were counted and correlated with donor age for all 11 HCMV proteins (B), lytic expressed proteins (C) and latent proteins (D). Spearman rank correlation (Spearman r_s (95% CI) and p values) results are indicated on each graph.

Supplementary Figure 4 – Identification of Donor ARIA012 results within cohort analyses

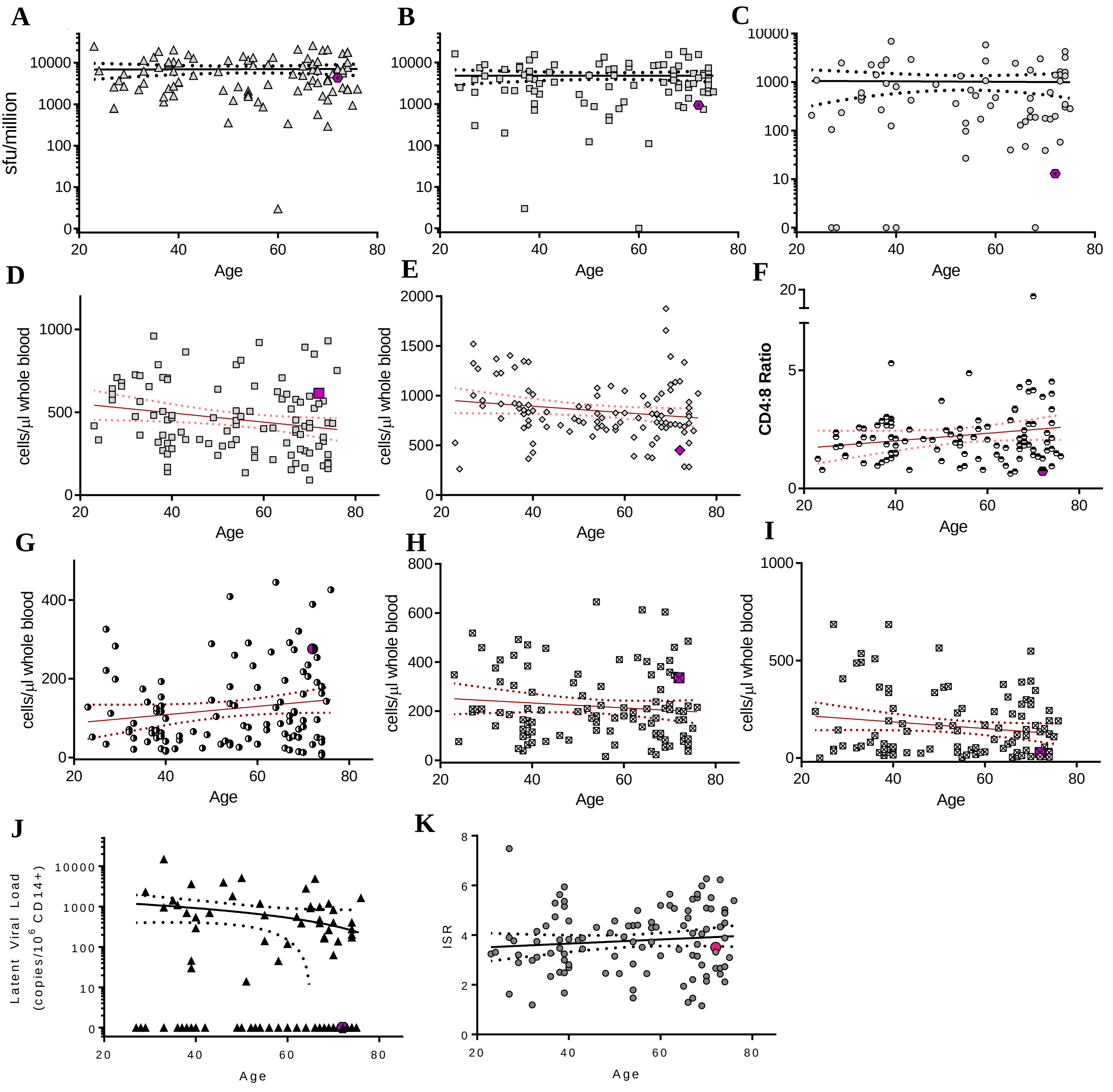

Supplementary Figure 4 – Identification of Donor ARIA012 within cohort analyses

Donor ARIA012 is identified in the above graphs as large magenta point, all graphs show measured parameter versus donor age. Shown is the total HCMV specific CD8+ IFNγ response (A), total HCMV specific CD4+ IFNγ response (B), total HCMV specific CD4+ IL-10 response (C) as spot forming units (sfu) per million. The absolute CD8+ T cell count (D), CD4+ T cell count (E) as cells/µl whole blood and the CD4:CD8 ratio (F). The differentiated CD8+ memory T_{EMRA} population (G), $CD28^{null}$ CD8+ T cell population (H) and $CD28^{null}$ CD4+ T cell population (I) as cells/µl whole blood. Lastly the latent viral load as copies/million CD14+ cells (J) and the total HCMV IgG as Immune Status Ratio (K) are shown.
